# Pesticides and parkinson’s disease: causal relationship at the population and individual level?

**DOI:** 10.1007/s00702-025-03054-3

**Published:** 2025-11-12

**Authors:** Matthias Höllerhage

**Affiliations:** Department of Neurology, Hannover Medical School, Carl-Neuberg-Str. 1, 30625 Hannover, Germany

**Keywords:** Parkinson’s disease, Pesticides, Occupational disease, Epidemiological studies

## Abstract

Pesticide exposure has been associated with an increased risk of Parkinson’s disease (PD). As a result, PD has been recognized as an occupational disease among agricultural workers in France for over a decade. In March 2024, a similar recommendation for recognition was issued in Germany. Pesticides encompass a wide range of functional classes and chemical groups, making it challenging–evaluate their specific effects. Numerous epidemiological studies, including cohort and case-control studies, have investigated the association between pesticide exposure and PD risk. While some report statistically significant associations, others yield inconclusive results, partly due–the inherent difficulties in accurately quantifying pesticide exposure and overlap in the use of different pesticides. In addition, a large body of experimental research has examined the neurotoxicity of various pesticides on dopaminergic neurons using both in vitro and in vivo models of PD. This review summarizes the findings from epidemiological and experimental studies and discusses the current evidence linking pesticide exposure–risk for Parkinson’s disease.

**Supplementary Information:**

The online version contains supplementary material available at https://doi.org/10.1007/s00702-025-03054-3.

## Introduction

 Pesticides represent a diverse group of chemical compounds employed for various purposes, primarily in agriculture and domestic environments. Broadly, they are categorized into plant protection products, which safeguard crops from pests and diseases, and biocidal products, which are employed in non-agricultural contexts - such as households-–control harmful organisms (e.g., disinfectants). The use of pesticides in human civilization dates back over 4000 years (Tudi et al. 2021).

In agriculture, pesticides are further classified by function into major groups including herbicides (targeting unwanted vegetation), fungicides (inhibiting fungal growth), and insecticides (targeting insects). These functional categories encompass numerous chemical classes, some of which overlap across uses or applications.

A growing body of epidemiological evidence suggests that chronic pesticide exposure may be a significant risk factor for the development of Parkinson’s disease (PD). Several studies have reported associations between occupational pesticide use and increased PD incidence or prevalence, indicating a possible etiological link. Notably, PD has been officially recognized as an occupational disease among agricultural workers in France since 2012. In March 2024, the German Federal Ministry of Labour and Social Affairs issued a recommendation–recognize “Parkinson-syndrome caused by pesticides” as an occupational disease.

The present review aims–summarize the available epidemiological data and the current evidence from preclinical PD models regarding the contribution of agricultural pesticides–the pathogenesis of PD. For the purpose of this review, the term “pesticide” will refer–plant protection products unless otherwise specified.

The first section outlines the principal functional categories and chemical classes of pesticides commonly used in agriculture. The second section reviews epidemiological studies examining the link between pesticide exposure and PD risk. The third section discusses experimental findings from in vitro and in vivo models investigating the effects of pesticides-induced effects on dopaminergic neurons and the nigrostriatal system. The final section outlines the regulatory status of pesticides linked–PD.

## Groups of pesticides

### Herbicides

Herbicides represent the most extensively used group of pesticides, accounting for approximately 47.5% of global pesticides usage (Pathak et al. 2022). Major chemical classes of herbicides include organophosphates (e.g. glyphosate, one of the most widely used herbicide worldwide), phenoxy acids, triazines, dinitroanilines, ureas, and amides. Additionally, paraquat is used as herbicide and has been extensively studies regarding an association–PD.

### Insecticides

Insecticides are the second most used group of pesticides, approximately accounting for 29.5% of global pesticide distribution (Pathak et al. 2022). Key chemical classes include organophosphates (e.g. chlorpyrifos), organochlorines (e.g. dieldrien), pyrethroids (e.g. permethrin), and neonicotinoids. Furthermore, rotenone, a naturally derived compound is one of the most extensively studied pesticides with regard–PD.

### Fungicides

Fungicides account for roughly 17.5% of global pesticide usage (Pathak et al. 2022). A major chemical class of fungicides are dithiocarbamates, such as maneb, which has also been extensively studied in the context of PD. Other important classes include azoles, particularly benzimidazoles (e.g. benomyl) and imidazoles. Dithiocarbamates, a class of organosulfur compounds, are extensively used as broad-spectrum fungicides and,–a lesser extent, as herbicides. First introduced in the 1930 s, they have become foundational in modern crop protection, especially for controlling fungal pathogens in high-value crops such as fruits, vegetables, and cereals (Thind and Hollomon 2018).

## Methods

To identify epidemiological studies that addressed a connection between pesticide exposure a systematic pubmed search was performed. Studies published between 1995 and the time of writing were considered using the following search string: ((“Parkinson Disease“[Mesh] OR parkinson*[tiab]) AND ((“Pesticides“[Mesh] OR pesticide*[tiab] OR herbicide*[tiab] OR insecticide*[tiab] OR fungicide*[tiab] OR organophosphate*[tiab] OR organochlorine*[tiab] OR carbamate*[tiab] OR paraquat[tiab] OR rotenone[tiab] OR maneb[tiab]) AND (“Epidemiologic Studies“[Mesh] OR “Case-Control Studies“[Mesh] OR “Cohort Studies“[Mesh] OR “Cross-Sectional Studies“[Mesh] OR epidemiolog*[tiab] OR “case-control“[tiab] OR “case control“[tiab] OR cohort[tiab] OR prospective[tiab] OR retrospective[tiab] OR “cross-sectional“[tiab] OR “population-based“[tiab] AND (“1995“[dp] : “2025“[dp]).–exclude reviews, meta-analysis, editorials, comments, and letters, the search string was extended with: NOT (Review[pt] OR Meta-Analysis[pt] OR Editorial[pt] OR Comment[pt] OR Letter[pt]).–further exclude basic research, the search string was refined with: AND humans[mh] NOT (Disease Models* [Mesh] OR neuroblastoma[tiab] OR rats[tiab] OR mice[tiab] OR cells[tiab] OR drosophila[tiab]). This search yielded 284 results. Titles and abstracts of these publications were systematically screened. Further exclusions were applied–eliminate: non-english publications, studies primarily focused on genetic PD or distinct genetic variants, studies investigating diseases other than PD (e.g. atypical parkinsonian syndromes or other neurodegenerative disorder), studies that did not focus on pesticides, studies that were considered as basic research (e.g. cell or animal model, biomarker studies), case-reports and case-series. In addition, one record without an abstract or full text and one publication not available online were excluded. After these steps, 76 publications remained and were systematically analyzed. Further exclusions were made for studies with very small patient groups (i.e. case-control groups with fewer than 50 patients), studies focused primarily on occupation rather than pesticide exposure, and studies lacking statistical analysis or appropriate control comparisons. The case-control-studies were then evaluated, using the GRADE criteria (Prasad 2024; Atkins et al. 2004; Guyatt et al. 2008).

## Results

### Epidemiological studies

Epidemiological studies from regions of the world have examined the potential relationship between agricultural activities and/or pesticide exposure and the risk of developing Parkinson’s disease. In the literature search, 54 studies were identified and analyzed. Of these, 37 were case-control studies and 17 cohort or registry studies that addressed a possible relationship between pesticide exposure and PD.

## Case control studies

The section summarizes case-control studies that investigated the association between pesticide exposure and PD risk. An overview of the these studies and their results is presented in Table 1. The studies are grouped as follows: First, case-control-studies with multiple publications derived from the same study population. Next, larger case control-studies including more than 200 PD cases, presented in descending order of case group size. Last, smaller case-control studies with fewer than 200 PD cases, also presented by case group size. The quality of evidence was assessed using the GRADE criteria, with ratings ranging from very low and moderate. The main limitation preventing higher ratings was inherently low baseline level from the case-control designs. Reasons for downgrading included risk of bias in single center studies and imprecisions caused by wide confidence intervals. Reasons for upgrading included large effect sizes (odds ratios [OR] > 2) or very large effect sizes (OR > 5), as well as trends toward dose-response relationships. A detailed summary of the GRADE assessment is provided in the Supplementary material.

**Table 1:** Case-control studies that investigated the association between pesticide exposure and PD risk

Study	Region	Subjects	Method	Main finding(s)	Comment	GRADE rating	Reference
Studies with multiple publications from the same study population							
Case-control studies from France with Members of the Agricultural Social Mutuality	France	Recruitment between 02/1998 and 08/1999 247 PD cases, 676 controls	Structured questionnaire	Increased risk for PD for gardening or professional pesticide use (OR 1.6; 95% CI 1.0–2.4), for professional only pesticide use (OR 1.8; 95% CI 1.1–3.1), trend for gardening pesticide use (OR 1.4; 95% CI 0.9–2.3) Increased PD risk for distinct functional groups (insecticides: OR 5.4; 95% CI 1.5–20.0; fungicides: OR 4.8; 95% CI 1.2–19.3)	Investigation of several factors (work on farm, exposure–pesticides, smoking	Low	Galanaud et al. 2005
		Recruitment between 02/1998 and 08/1999 227 PD cases, 577 controls	Self-questionnaires	Significantly Increased PD risk for distinct functional groups: insecticides: OR 5.4; 95% CI 1.5–20.0; fungicides: OR 4.8; 95% CI 1.2–19.3 in men older than 65 years with high cumulative exposure. Increased risk for specific chemical groups: organochlorides (OR 2.4; 95% CI 1.2–5.0), amides (OR 3.1; 95% CI 1.2–8.3), dithiocarbamates (OR 2.1; 95% CI 1.0–4.3) in all men and for organochlorides (OR 4.2; 95% CI 1.5–11.9), nitriles (OR 11.4; 95% CI 1.4–93.7), and phenoxy pesticides (OR 2.9; 95% CI 1.1.–7.3) in men older than 65 years	Same study population as in Galanaud et al. 2005 stratification by age in male participants functional pesticide groups and chemical groups only investigated in male participants.	Low	Elbaz et al. 2009
		Residents in MSA database (2006-07) who bought at least one anti-parkinsonian drug 133 PD male cases, 298 male controls	self-administered questionnaire	Increased risk for tremor-dominant PD in medium (OR 2.75; 95% CI 1.06–7.13) and high (OR 4.13; 95% CI 1.53–11.14) exposure groups, increased risk of non-tremor-dominant PD in medium exposure group (OR 2.76; 1.02–7.52) and trend in high exposure group (OR 2.26; 95% CI 0.77–6.64) Trend towards increased risk for tremor-dominant PD with high herbicide exposure (1.45; 95% CI 0.71–3.00), but not for non-tremor dominant PD (OR 0.68; 95% CI 0.30–1.56) Increased risk for tremor-dominant PD with high insecticide exposure (OR 2.58; 95% CI 1.23–5.40), but not for non-tremor dominant PD (OR 1.08; 95% CI 0.48–2.43) Increased risk for tremor-dominant PD with high fungicide exposure (OR 2.83; 95% CI 1.11–7.23), trend for non-tremor dominant PD (OR 1.67; 95% CI 0.60–6.62)	Exclusion of PD patients > 80 years and/or > 15 years disease duration Only male participants. Stratification of exposure in duration and cumulative exposure Stratification by tremor-dominant and non-tremor-dominant PD	Low	Moisan et al. 2015
Farming and Movement Evaluation (FAME), nested within the Agricultural Health Study (AHS)	Iowa and North Carolina, US	Recruitment between 1993 and 1997 110 PD cases, 358 controls	Computer-assisted telephone interviews, questions for 31 selected pesticides	Significant increased PD risk for paraquat (OR 2.5; 95% CI 1.4–4.7) and rotenone (OR 2.5; 95% CI 1.3–4.7) users	Case-control study within the AHS. Population of mostly farmers and their spouses	Moderate	Tanner et al. 2011
		Recruitment between 1993 and 1997 69 PD cases, 267 controls	Structured telephone interviews between 2002 and 2008	Significantly increased PD risk with exposure–paraquat (OR 2.6; 95% CI 1.1–6.1) and rotenone (OR 5.5; 95% CI 2.0–15.3). No increased PD risk with exposure–permethrin (OR 1.2; 95% CI 0.5–3.9) or trifuralin (OR 0.9; 95% CI 0.4–2.1)	Subset of the population from Tanner et al. 2011 Investigation of the effect of protective gloves Investigation of exposure–paraquat, permethrin, rotenone, and trifuralin	Moderate	Furlong et al. 2015
Parkinson Environment Gene (PEG) study	Rural communities in California, US	Newly diagnosed PD cases between 01/1998 and 01/2007 368 cases, 341 controls	Telephone interview	Increased PD risk associated with use of paraquat *and* maneb (OR 2.14; 95% CI 1.24–3.68), but only when exposed between 1974 and 1999, exposure after 1990 not associated with elevated PD risk (OR 0.93; 95% CI 0.45–1.94). Paraquat only exposure not associated with increased PD risk (OR 1.01; 95% CI 0.71–1.43)	Study population originally described in Kang et al. 2005 In this study: investigation of residential exposure–maneb and/or paraquat	Moderate	Costello et al. 2009
		Newly diagnosed PD cases between 01/1998 and 01/2007 368 PD cases, 341 controls	Telephone interview	increased PD risk associated with exposure zo diazinon (organophosphate); OR 1.58; 95% CI 1.03–2.43 Other pesticides with trends–increased risk: dimethoate, methomyl, chlorpyrifos, propargite, paraquat	Same study population as in Costello et al. 2009 estimated pesticide exposure from well water concentrations in the different counties in the study area	Very low	Gatto et al. 2009
		Newly diagnosed PD cases between 01/1998 and 01/2007 362 PD cases, 341 controls	Telephone interview	Residential *and* occupational use of ziram (OR 3.01; 95% CI 1.69–5.38) or paraquat (OR 1.5; 95% CI 1.03–2.18) associated with increased PD risk, not with use only in one setting Residential (OR 1.71; 95% CI 1.06–2.77) or occupational (OR 1.71; 95% CI 1.06–2.77) use of maneb or both (OR 2.26; 95% CI 1.22–4.20) associated with significantly increased PD risk	Same study population as in Costello et al. 2009 Investigation of ambient exposure–several pesticides (paraquat, maneb, and ziram)	Moderate	Wang et al. 2011
		Newly diagnosed PD cases between 01/2001 and 01/2007 Controls between 2002 and 2011 357 PD cases, 754 controls	Interview	Increased PD risk associated with paraquat exposure (OR 1.43; 95% CI 1.11–1.84)	Same cases as in Costello et al. 2009; control groups extended Focus on traumatic brain injury and paraquat exposure with regard–PD risk Paraquat exposure evaluated combined as residential and workplace exposure	Low	Lee et al. 2012
Parkinson Environment Gene (PEG) study *(continued)*	Rural communities in California, US	Newly diagnosed PD cases between 01/2001 and 01/2007 Controls between 2002 and 2011 357 PD cases, 807 controls	Interview, self-reported	Ever household use of pesticides associated with increased PD risk (OR 1.47; 95% CI 1.13–1.92) Household use of organophosphates associated with significantly increased PD risk ((OR 1.71; 95% CI 1.21–2.41), specifically chlorpyrifos (OR 2.73; 95% CI 1.03–7.24)	Same cases as in Costello et al. 2009; control groups extended as in Lee et al. 2012 Focus on household-use of different pesticides (organophosphates, organothiophosphates) Specific investigation of the exposure–chlorpyrifos and diazinon	Low	Narayan et al. 2013
		Newly diagnosed PD cases between 01/2001 and 01/2007 Controls between 2002 and 2011 357 PD cases, 750 controls	Development of a job exposure matrix (JEM) Different models–calculate exposure in quartiles (no, low, medium, high).	In adjusted model, increased risk for PD in the medium (OR 1.61; 95% CI 1.00–2.62) and high exposure group (OR 1.97; 95% CI 1.11–3.48), but not in low exposure group (OR 1.17; 95% CI 0.61–2.24)	Same cases as in Costello et al. 2009; control groups extended as in Lee et al. 2012 Investigation of occupational pesticide exposure using a job exposure matrix (JEM) No distinction between different types of pesticides Stratification by exposure intensity Use of different models for adjustment	Moderate	Liew et al. 2014
		Newly diagnosed PD cases between 01/2001 and 01/2007 Controls between 2002 and 2011 357 PD cases, 752 controls	Investigation of pesticide use reports, land-use maps and geocoded address information	Increased risk with exposure–several organophosphates Workplace exposure for more organophosphates associated with significantly increased PD risk compared–residence only exposure. Highest risk for the different exposure paradigms: workplace only: demeton (OR 4.74: 95% CI 2.27–9.92); residential only: merphos (OR 3.13; 95% CI 1.67–5.79); residential and workplace: demeton (OR 5.93; 95% CI 1.69–20.87)	Same cases as in Costello et al. 2009; control groups extended as in Lee et al. 2012 Investigation of ambient exposure–multiple organophosphates (residence and workplace) For some pesticide small exposure numbers	Moderate	Wang et al. 2014
		Newly diagnosed PD cases between 01/2001 and 01/2007 Controls between 2002 and 2011 360 cases, 827 controls	Telephone interview	Occupational use of any pesticide associated with significantly increased PD risk (OR 2.5; 95% CI 1.5–4.15) Increased with PD risk with use of herbicides (OR 2.45; 95% CI 1.37–4.36), insecticides (OR 2.1; 95% CI 1.22–3.60), and fungicides (OR 3.11; 95% CI 1.65–5.88) Increased PD risk with exposure–carbamates (OR 5.55; 95% CI 1.81–17.04)	Same cases as in Costello et al. 2009; control groups extended as in Lee et al. 2012 Investigation of household, ambient residential, and ambient occupational pesticide exposure as well as actual use of pesticides, Stratification by functional groups	Moderate	Narayan et al. 2017
		829 PD cases (wave 1: 2000–2007; wave 2: 2009–2015); 824 controls	Pesticide use reports and pesticide application data	Trend towards increased PD risk for men (OR 1.27; 95% CI 0.97–1.70) and women (OR 1.19; 95% CI 0.86–1.66) with any occupational exposure–paraquat between 1974 and year of inclusion No increased PD risk for men with any residential exposure–paraquat (OR 1.00; 95% CI 0.74–1.34), trend towards increased PD risk for women with any residential exposure–paraquat (OR 1.22; 95% CI 0.88–1.71) between 1974 and year of inclusion	Recruitment in 2 waves (wave 1: same cases as in Costello et al. 2009) Distinct investigation of paraquat use Stratification by residence and occupational use, time period of exposure and sex Adjustment of use of other pesticides	Low	Paul et al. 2024
Case-control study from the Netherlands	Netherlands	Recruitment between 04/2014 and 06/2012) cases diagnosed PD between 01/2006 and 12/2011 444 PD cases, 876 controls	Standardized computer-assisted telephone interview Different algorithms–estimate exposure, including a job exposure matrix	In the JEM approach correlation coefficients between 0.73 and 0.85 between the exposure–different functional classes of pesticides (herbicides, fungicides, insecticides) Trend towards increased PD risk in highest cumulative exposure group–any pesticide (OR 1.36; 95% CI 0.89–2.09) Trends towards increased PD risk in highest exposure groups for insecticides (OR 1.48 95% CI 0.90–2.41), herbicides (OR 1.26; 95% CI 0.71–2.24), and fungicides: 1.26; 95% CI 0.76–2.07) Increased PD risk associated with high exposure–benomyl (OR 2.46; 95% CI 1.16–5.22) No significant association between the exposure–other pesticides with PD risk	Comprehensive selection of participants in 5 hospitals, because in the Netherlands all PD patients receive hospital care. Investigation of correlations between the different pesticide exposures Stratification of exposure in never, low, medium, and high Investigation of different functional groups and individual pesticides	Low	van der Mark et al. 2014
		Recruitment between 04/2014 and 06/2012) cases diagnosed PD between 01/2006 and 12/2011 352 PD cases, 607 controls	Telephone interview	Significantly increased PD risk in the vicinity of exposure–several of the selected pesticide, especially in the highest exposure groups Highest PD risk associated with fenpropimorph (OR 2.79; 95% CI 1.33–5.89) Trends towards an increased PD risk for other pesticides, e.g. paraquat (OR 1.46; 95% CI 0.95–2.23), lindane (OR 1.11; 95% CI 0.73–1.68), maneb (OR 1.23; 95% CI 0.80–1.90), benomyl (OR 1.54; 95% CI 0.47–5.04)	same cases as in van der Mark et al. 2014 Investigation of environmental exposure–pre-selected pesticides stratified by vicinity of residence–place of pesticide use Stratification by intensity of exposure No distinction between different pesticides	Low	Brouwer et al. 2017
Group Health Cooperative	Western Washington State	Incident PD cases between 1992 and 2002 156 PD cases, 241 controls	structured interview	No increased PD risk with occupational pesticide exposure: OR 1.01; 95% CI 0.53–1.92 (any pesticide), OR 1.41; 95% CI 0.51–3.88 (herbicides), OR 0.88; 95% CI 0.44–1.76 (insecticides), OR 0.38; 95% CI 0.07–2.05 (fungicides). No increased PD risk with home-based pesticide exposure: OR 0.95; 95% CI 0.66–1.37 (any pesticide), OR 1.41; 95% CI 0.51–3.88 (herbicides), OR 0.88; 95% CI 0.44–1.76 (insecticides), OR 0.55; 95% CI 0.29–1.05 (fungicides)	distinction between occupational and home-based exposure distinction between different functional groups (herbicides, insecticides, fungicides) and for occupational exposure of different compounds (organophosphates, parathion, diazinon, malathion, paraquat)	Very low	Firestone et al. 2005
		Incident PD cases between 1992 and 2006 404 PD cases, 526 controls	structured interview	No significant associated between pesticide exposure and PD in male: OR 0.6; 95% CI (0.3–1.29) or in female: OR 3.9 (95% CI 0.39–39.4) persons	Extension of the study population described in Firestone et al. 2005 Investigation of several occupations Very low number of persons with pesticide exposure, especially in subgroup of female persons (male: *N* = 12 in cases and *N* = 24 in controls; female: *N* = 3 in cases and *N* = 1 on controls)	Very low	Firestone et al. 2010
Large case-control studies with more than 200 PD cases (sorted by case number)							
Geoparkinson study	5 regions across Europe: Scotland, southeastern Sweden, northern Italy, eastern Romania, Malta	Recruitment between 06/2000 and 09/2004 767 PD cases, 1989 controls	interviewer-administered questionnaire JEM–estimate exposure	increased risk for PD with high pesticide exposure: low exposure: OR 1.09; 95% CI 0.77–1.55, high exposure: 1.39; 95% CI 1.02–1.89	Investigation of multiple environmental factors Stratification in low and high exposure No distinction between different pesticides	Low	Dick et al. 2007
Case-control study from Italy	From 6 neurology departments in Italy;	Recruitment between 09/2018 and 09/2019 694 PD cases, 640 controls	Semi-structured questionnaire	Increased risk for PD with pesticide exposure (OR 2.99; 95% CI 1.8–5.1)	Investigation of multiple life style factor No distinction between different types of pesticides	Moderate	Belvisi et al. 2020
Case-control study from Pakistan	Lady Reading Hospital, Peshawar, KPK, Pakistan	Recruitment between 01/2017–01/2018 600 PD cases, 1200 controls	Questionnaire	Increased risk for PD with pesticide use (OR 4.52; 95% CI 2.49–8.19) Increased risk for PD with use of aldrin (organochloride), OR 11.0; 95% CI 4.02–30.07)	Distinction between different pesticides, only aldrin with significantly increased OR	Very low	Tufail et al. (2019)
Case-control study from the United States and Canada	California, South Florida, Georgia, Texas, New York, Ontario (CAN), Kansas, Pacific Islands	Recruitment between 07/2004 and 05/2007 519 PD cases, 511 controls	Standardized computer-assisted telephone interviews	increased risk for PD with pesticide use: OR 1.90; 95% CI 1.12–3.21) increased risk for PD with use of 2,4-dichlorophenoxyacetic acid: OR 2.59; 95% CI 1.03–6.48) other pesticides n.s due–low numbers.	In addition–pesticides without distinction, 8 specific pesticides were asked. Most of them had (very) low exposure numbers: 2,4-dichlorophenoxyacetic acid (16 cases; 7 controls), paraquat (9;4), permethrin (7;2), dieldrin (3;2), diquat (1;1), maneb (0;0), mancozeb (1;1), rotenone (1;1).	Low	Tanner et al. 2009
Case-control study from Germany	Germany	Recruitment of cases with diagnosis of PD 1987 or later 380 PD cases, 376 neighborhood controls, 379 regional controls	Own assessment of ever/never exposure–several neurotoxic compounds	Significantly increased PD risk associated with herbicide (OR 2.4; 95% CI 1.0–6.0) and trend with insecticide (OR 2.1; 95% CI 0.9–4.8) use compared–regional controls, trend for increased PD risk when compared–neighborhood controls for herbicides (OR 2.2; 95% CI 0.9–5.2) and insecticides (OR 1.6; 95% CI 0.7–3.4)	Shows regional effect of pesticide use Distinction between herbicides and insecticides, and between organochlorines and alkylated phosphates/carbamates (the 2 latter in one group).	Low	Seidler et al. 1996
Case control study from North Carolina	North Carolina	Recruitment from 2000–2006 319 PD cases, 296 controls	Structured telephone interview	Increased PD risk with a history of pesticide use (OR 1.61; 95% CI 1.13–2.29), only in cases with negative family history for PD	Family-bases case-control study 252 of the controls were relatives of cases.	Low	Hancock et al. 2008
Case-control study from Australia	South-East Qld and Central New South Wales, Australia	No information on recruitment period 224 PD cases, 310 controls	Risk factor questionnaire	Not significantly increased PD risk with exposure–herbicides and pesticides: OR 1.2; 95% CI 0.8–1.5 Significantly increased PD risk with rural residency (OR 1.8; 95% CI 1.7–2.5) in the univariate analysis confounded by an inverse correlation between hypertension and PD.	Exposure–herbicides and pesticides used as one variable without further distinction	Very low	McCann et al. 1998
Case-control study from Hong Kong	Hong Kong	No information on recruitment period 215 PD cases, 313 controls	Questionnaire focusing on possible risk factors for PD	Not significantly Increased risk for PD with pesticide exposure in farming in the univariate analysis: OR 1.80; 95% CI 0.903–3.58 Increased PD risk with pesticide exposure in women (OR 6.84; 95% CI 1.90–24.7), but not in men (OR 0.68; 95% CI 0.25–1.83)	Investigation of several factors, including smoking, pesticide exposure, well water drinking, and farming	Very low	Chan et al. 1998
Smaller case-control studies with less than 200 PD cases (sorted by cases)							
Case-control study from India	Eastern India	Recruitment between 01/2006 and 12/2009 175 PD cases, 350 controls	Interview with structured questionnaire	Increased risk for PD with pesticide exposure: OR 17.4; 95% CI 4.97–58.84	No distinction between different types of pesticides	Very low	Sanyal et al. 2010
Rochester Epidemiology Project	Olmsted County, Minnesota,	Diagnosis of PD between 1976 and 1995 149 PD cases, 129 controls	Proxy interview with structured questionnaire	Not significantly increases PD risk with pesticide exposure in whole case group: OR 1.5; 95% CI 0.8–2.9) Increased risk for PD in male persons: OR 2.4; 95% CI 1.1–5.4 In female: OR 0.6; 95% CI 0.2–1.9	Investigation of several occupational toxins Stratification of pesticides in herbicides and insecticides only in male persons due–low female overall case numbers (*N* = 6)	Low	Frigerio et al. 2006
Case-control study from New York	Recruited in New York City, US	Distribution of survey between 01/2016 and 07/2016 149 PD cases, 105 controls	Paper survey	Investigation of multiple life style factor Significantly increased risk for PD with pesticide exposure (OR 2.84; 95% CI 1.34–6.00)	Investigation of several exposures (i.e. smoking, alcohol, pesticides, medication) No distinction between different types of pesticides	Very low	Shermon et al. 2022
Henry Ford Health System (HFHS)	Tricounty metropolitan Detroit area	Recruitment between 04/1991 and 07/1995 144 PD cases, 464 controls	Face-to-face interview with risk factor questionnaire	Increased PD risk with exposure–herbicides (OR 4.1; 95 W% CI 1.37–12.24) and insecticides (OR 3.55; 95% CI 1.75–7.18) Not significantly increases PD risk with exposure–fungicides (OR 1.60; 95% CI 0.47–5.45)	Differentiation between different functional classes (herbicides, insecticides, fungicides) Differentiation between occupational use on farm and in gardening as a hobby	Low	Gorell et al. 1998
Case control-study from Canada	Calgary, Canada	PD diagnosis between 01/1984 and 12/1987 130 PD cases, 260 controls	Personal interviews and proxy-derived data	Increased risk for PD associated with herbicide use (OR 2.36; 95% CI 1.10–5.04)	Investigation of several factors (e.g. herbicide us, family history of PD or essential tremor, head trauma, and smoking)	Low	Semchuk and Love (1995)
Case-control study from Taiwan	Taiwan	Enrollment between 07/1993 and 06/10,995 120 PD cases, 240 controls	Structured open-ended questionnaire	Significantly increased PD risk associated with rural residence (OR 2.04; 95% CI 1.23–3.38) and farming (OR 1.81; 95% CI 1.25–2.64) Significant association between herbicide/pesticide use (OR 2.89; 95% CI 2.28–3.66) and paraquat use (OR 3.22; 95% CI 2.41–4.31) and PD risk	Investigation of multiple environmental factors, including herbicide/pesticide use and paraquat use	Low	Liou et al. 1997
Case-control study from Serbia	Belgrade, Serbia	Enrollment between 01/2001 and 11/2005 110 PD cases, 220 controls	Interview with structured questionnaire	Increased risk for PD with insecticide exposure (OR 3.22; 95% CI 1.32–7.87)	Investigation of several environmental factors No distinction between different types of insecticides	Low	Vlajinac et al. 2010
Case-control study from Brazil	Rio de Janeiro, Brazil	Enrollment between 01/1996 and 02/2007 92 PD cases, 110 controls	Questionnaire	Trend towards increased risk for PD with exposure–herbicides/insecticides: OR 2.49; 95% CU: 0.53–13.14	Question about regular handling of herbicides or insecticides, no further distinction.	Low	Werneck et al., 1999
Case-control study from Brazil	State of Mato Grosso, Brazil	Enrollment between 07/2017 and 08/2018 88 PD cases, 284 controls	Structured questionnaire	Significantly increased PD risk associated with direct management of pesticides at workplace (OR 3.78; 95% CI 1.92–7.45) and history of exposure–pesticides at workplace (OR 2.35; 95% CI 1.36–4.06)	Investigation of occupational and environmental exposure–pesticides No distinction between different types of pesticides	Very low	Silvestre et al. 2020
Case-control study from Southwestern France	Southwestern France, PAQUID cohort	Enrollment between 11/1997 and 07/1999 84 PD cases, 252 controls	Assessment of likelihood–pesticide exposure by occupational calendar Use of a job exposure matrix	Significantly increased PD risk associated with occupational pesticide exposure (OR 2.2; 95% CI 1.11–4.34)	Investigation of several lifestyle factors (e.g. marital status, smoking, rural residence, vineyard residency No distinction between different types of pesticides	Very low	Baldi et al. 2003a
Case-control study from East Texas	East Texas	No information on recruitment period 73 PD cases, 71 controls	Standardized questionnaire	No significant association of overall pesticide exposure with PD risk No significant association with use of herbicides (OR 1.1; 95% CI 0.6–2.0), insecticides (OR 1.4; 95% CI 0.4–4.4), or fungicides (OR 1.4; 95% CI 0.4–4.4) Increased PD risk associated with rotenone (OR 10.0; 95% CI 2.9–34.3) and chlorpyrifos (OR 2.0; 95% CI 1.02–3.8) use	Investigation of several lifestyle factors (e.g. marital status, smoking, alcohol and caffeine intake) Investigation of exposure–several environmental and occupational factors Distinction between different pesticide containing products, some with very low numbers of exposed persons	Very low	Dhillon et al. 2008

### French case-control-studies, including members of the agricultural social mutuality

Several French case-control studies have investigated pesticide exposure and PD risk, recruiting members of the Agricultural Social Mutuality (in French: Mutualité Sociale Agricole; MSA), where healthcare for PD is provided free of charge. In one study, MSA members who applied for free PD-related healthcare between February 1998 and August 1999 were recruited as cases and matched–MSA members who requested reimbursement for other health conditions. Pesticide exposure was assessed via questionnaires and home interviews conducted by occupational physicians (Galanaud et al. 2005; Elbaz et al. 2009). In this analyis, 247 PD cases were matched–676 controls. Professional pesticide use was associated with an increased PD risk (OR 1.8; 95% CI 1.1–3.1) compared with never users (Galanaud et al. 2005).

A follow-ups analysis of the same study population included 224 PD cases and 577 controls, focusing on exposure–different functional groups of pesticides. Interestingly, professional exposure–insecticides increased PD risk in men, while professional exposure–fungicides increased PD risk in women. In men over 65 years, higher cumulative exposure, defined as exposure above the median,–insecticides and fungicides was associated with increased PD risk (insecticides: OR 5.4; 95% CI 1.5–20.0; fungicides: OR 4.8; 95% CI 1.2–19.3). No significant increase was observed in men under 65, and the sample size was too small for age-stratification in women. Herbicide exposure was not significantly associated with increased PD risk in either sex. Analysis by chemical family revealed significant associations in men for organochlorides (OR 2.4; 95% CI 1.2–5.0), amides (OR 3.1; 95% CI 1.2–8.3), and dithiocarbamates (OR 2.1; 95% CI 1.0–4.3). Among men over 65, ssociations were even stronger for organochlorides (OR 4.2; 95% CI 1.5–11.9), nitriles (OR 11.4; 95% CI 1.4–93.7), and phenoxy pesticides (OR 2.9; 95% CI 1.1–7.3) (Elbaz et al. 2009).

In another French case-control study, involving only male MSA members, 133 farmers, who purchased antiparkinsonian drugs in the years 2006 and 2007, were matched–298 farmers without PD. The analysis was stratified by tremor-dominant and non-tremor-dominant PD. Although overall pesticide exposure time did not differ significantly between cases and controls, cumulative exposure (i.e. total number of applications) and average annual applications were significantly associated with PD risk. Subanalyses showed that fungicide use and higher average exposure intensity–insecticides were linked–increased PD risk, however only for tremor-dominant PD. By contrast, herbicide exposure was not significantly associated with PD risk (Moisan et al. 2015).

### The farming and movement evaluation (FAME) study

The FAME study, a case-control-study that was nested within the Agricultural Health Study (AHS), investigated pesticide exposure among 110 PD cases and 358 healthy controls who reported pesticide use (Tanner et al. 2011). The study found significant associations between PD and use of paraquat (OR 2.5; 95% CI 1.4–4.7) and rotenone (OR 2.5; 95% CI 1.3–4.7). A follow-up study in the same study population examined protective glove use among individuals involved in pesticide mixing or application. After adjustment for smoking, sex, age, and hygiene measures, including glove use, paraquat (OR 2.6; 95% CI 1.1–6.1) and rotenone (OR 5.5; 95% CI 2.0–15.3) use remained significantly associated with PD risk compared–never use (Furlong et al. 2015). The level of evidence for both publications was moderate, justified by the large effect sizes.

### The Parkinson environment gene (PEG) study

The PEG study, conducted in rural communities in California, generated nine publications identified in the literature search. Initially, 162 PD cases diagnosed between 1998 and 2003 were investigated and matched–controls (Kang et al. 2005). The study was later expanded–include PD cases diagnosed between 1998 and 2007, yielding 1167 PD cases, with case groups of ~ 360 in earlier publications and ~ 750 in later ones (Table 2).

**Table 2:** Cohort studies and registries that investigated investigated the association between pesticide exposure and PD risk.

Study	Type	Region	Individuals	Methods	Main finding(s)	Comment	References
PAQUID	Cohort	Southwestern France	Persons above 65 in the year 1987 1,507 participants, 320 with pesticide exposure	occupational exposure assessed by questionnaire	Increased PD risk associated with occupational pesticide use, but only in male participants (OR 5.63; 95% CI 1.47–21.58), not in female (OR 1.02; 95% CI 0.11–4.82)	More female (922) participants than male (585).	Baldi et al. 2003b
AGRICAN, Mutualité Sociale Agricole (MSA)	Cohort	France	Enrollment between 2005–2007 Individuals above 18 years, living in one of 11 French areas in 2004 Self-reported PD: 1,732 cases, 148,078 controls	French crop-exposure matrix (CEM) PESTIMAT used–assess exposure investigation of PD association with different forms of farming and report of pesticide exposure investigation of ever version never exposure–several pesticides investigation of association of PD–duration of use of several pesticides	Increased PD risk for persons reporting pesticide poisoning (OR 1.51; 95% CI 1.14–1.99 for once poisoning; OR 2.25; 95% CI 1.52–3.38 for several times poisoning) Increased PD risk for exposure–several dithiocarbamates (ORs 1.31–1.52; 95% CI from 1.08–1.60–1.26–1.83), rotenone (OR 1.57; 95% CI 1.08–2.29), paraquat (OR 1.43; 95% CI 1.17–1.75), and diquat (OR 1.39; 95% CI 1.14–1,68)	Large cohort of persons with retired or active occupation in agriculture	Pouchieu et al. 2018
Mutualité Sociale Agricole (MSA), French National Health Insurance database	Registry	France	Persons that received reimbursement for antiparkinsonian drug between 2009 and 2015 Incident PD cases in farmers (2010–2015) 10,282 PD cases	Investigation of expenditures for pesticides in France stratified by farming type	higher PD incidence in areas with vineyards with high expenditures for pesticides IRR = 1.16, 95% CI 1.06–1.28 trend towards increased incidence in men in the sex stratified analysis no increased incidence in men in the sex stratified analysis no significant effect on PD incidence by pesticide expenditures by other types of farming	Large registry covering most of the French population	Perrin et al. 2021
TRACTOR, members of Mutualité Sociale Agricole (MSA), French Health insurance in agriculture	Cohort	France	Recruitment between 2002 and 2016 1,088,561 farm managers in total, 8,845 PD cases	Investigation of reports of farming activities–the MSA and request for reimbursement of PD drugs	Increased risk for PD associated with distinct forms of farming (pig farming, dairy farming, mixed cattle farming, and crop farming)	Included here, because it is one of the largest and most comprehensive cohort studies currently available. No assessment of pesticide exposure itself.	Petit et al. 2025
Italian Longitudinal Study on Aging (ILSA)	Cohort	Italy, eight study centers	Inhabitants of the eight study areas aged 65–84 in March 1992 4,496 persons screened for PD and risk factors 113 PD subjects, 4,383 non-PD subjects	screening of a subset of a population for PD investigation of several risk factors, including pesticide-use license	Increased PD risk for persons with a pesticide-use license (OR 3.68; 95% CI 1.57–8.64)	Small number of persons with licenses (7 PD subjects, 83 non-PD subjects)	Baldereschi et al. 2003
Netherlands Cohort Study on diet and cancer	Registry	Netherlands	120,852 persons, aged 55–69 in 1986, follow-up until 2003 402 deaths related–PD in men and 207 in women	questionnaire on occupational history ALOHA + JEM–estimate occupational exposure–pesticides investigation of deaths in persons with PD diagnosis	Trend towards increased PD mortality in men with pesticide exposure, in low exposure group: HR: 1.35; 95% CI 0.81–2.26), in high exposed group: HR: 1.27; 95% CI 0.86–1.88 In cumulative exposure analysis significantly increased PD mortality in shortest exposure tertile: HR: 1.89; 95% CI 1.11–3.22, but not in longer exposed quartiles	Analysis of data obtained with male persons Higher PD mortality associated with smaller cumulative occupational exposure–pesticides	Brouwer et al. 2015
Agricutural Health Study (AHS)	Cohort	Iowa and North Carolina, US	Enrollment between 1993 and 1997 Follow-up between 1999 and 2003 (phase 2), 79,557 individuals total, 55,931 at follow up, 83 prevalent PD cases, 78 incident PD cases	Self-administered enrollment questionnaires Computer-assisted follow-up telephone interviews	Trend towards association of ever pesticide use and incident PD (OR 1.3; 95% CI 0.5–3.3) Trend towards negative association between ever pesticide use and prevalent PD (OR 0.5; 95% CI 0.2–1.1) Higher use of paraquat (OR 1.8; 95% CI 1.0–3.4) and cyanazine (OR 2.6; 95% CI 1.4–4.9) in incident PD Higher use of trifluralin (OR 1.7; 95% CI 1.0–3.2) and 2,4,5-Trichlorophenoxyacetic acid (OR 1.8; 95% CI 1.0–3.3) in prevalent PD No significant associations between incident or prevalent PD with insecticide and fungicide use	Large cohort study Distinction between incident and prevalent PD	Kamel et al. 2007,
Agricutural Health Study (AHS)	Cohort	Iowa and North Carolina, US	Enrollment between 1993 and 1997 (phase 1), follow-up between 1999 and 2003 (phase 2), 2005 and 2010 (phase 3), and 2013 and 2016 (phase 4) 38,274 pesticide applicators, 27,836 spouses 373 new PD cases in applicators, 118 in spouses	Self-administered enrollment questionnaires Computer-assisted follow-up telephone interviews (phases 2 and 3) Computer-assisted follow-up telephone interviews or self-administered questionnaire (phase 4)	Trend towards higher PD incidence in pesticide applicators for higher use quartiles (3rd quartile: HR: 1.27; 95% CI 0.82–1.98; 4th quartile: HR: 1.07; 95% CI 0.69–1.67)	Investigation of several lifestyle factors Investigation of a large list of pesticides Comparison between pesticide applicators and their spouses	Shretha et al. 2020
State Department of Health Washington	Cohort	Washington State, US	Recalled particants, originally enrolled between 1972 and 1976 323 participants, 238 with pesticide exposure, 72 non-exposed	Recall of participants in the Washington State Department of Health cohort study Self-administered questionnaire on pesticide exposure Calculation of PD prevalence related–the exposure–several pesticides	No association between use of well water, or farm employment with PD prevalence No association between any exposure–several pesticides with increased PD prevalence In the analysis of exposure years in tertiles, significantly increased prevalence ratios for PD in longest exposure–any pesticide (adjusted PR: 2.0 95% CI 1.0–4.2) Trends for increased prevalence in group of highest exposure–insecticides (adjusted PR: 1.7; 95% CI 0.9–3.3) and herbicides (adjusted PR: 1.7; 95% CI 0.9–3.2) No increased prevalence in group of highest exposure–fungicides (adjusted PR: 0.7; 95% CI 0.7–1.5)	Report of PD prevalence ratios associated with exposure–use of well water, farm employment, and several pesticides	Engel et al. 2001
Registry study from the Washington State Department of Health	Registry	Washington State, US	4,591 PD related deaths (2011–2015), 659 premature	Pesticide application land use classifications	Increased risk for premature mortality in PD associated with glyphosate exposure (OR 1.33; 95% CI 1.06–1.67)	Analysis of premature mortality (before age of 75 years) in PD patients.	Caballero et al. 2018
California Vital Statistics	Retrospective analysis of a registry	California, US	7,516 cases with PD as underlying cause of death between 1984 and 1994 15,222 with PD as associated cause of death between 1994 and 1993 Control group of 498,461 cases with ischemic heart attack between 1984 and 1994	Investigation of pesticide-use reports from 1972–1990 Stratification by no, low, moderate, and high pesticide use in different counties Stratification by death between 1984 and 1988 and between 1989 and 1994 Calculation of duration of residence in the county	Increased prevalence odds ratio for PD as underlying cause of death in counties with the highest pesticide use (death from 1989–1994: POR 1,45; 95% CI 1,32–1,59, from 1984–1988: POR 1.19; 95% CI 1.06–1.34)	Retrospective analysis of deaths related–PD and estimation of their lifetime pesticide exposure	Ritz et al., 2000
Cancer Prevention Study II Nutrition Cohort	Cohort	United States of America	Initiated in 1992, follow-up in 1997, 1998, and 2001 143,325 subjects that returned 2001 survey, 7,864 exposed–pesticides, 340 PD cases	Survey with questions about exposure–pesticides/herbicides	Increased PD risk after pesticide exposure (RR: 1.8; 95% CI 1.3–2.5)	Originally designed–investigate lifestyle factors associated with cancer.	Ascherio et al. 2006
Colorado Medicare beneficiaries	Registry	Colorado, US	332,971 Medicare beneficiaries 4,207 prevalent PD cases	investigation of pesticide concentration in ground water and association–PD calculation of PD risk dependent of ground water pesticide concentration	significant association between overall pesticide concentrations in ground water and PD prevalence (OR 1.03; 95% CI 1.02–1.04) significant association between atrazine concentrations in ground water and PD prevalence (OR 1.04; 95% CI 1.03–1.05) 3% increased PD risk for every 0.01 mg/L pesticide concentration and 4% increased PD risk for every 0.01 mg/L atrazine concentration	Direct measurement of pesticide concentrations in ground water Data not dependent on exposure questionnaires	James and Hall 2015
Comprehensive Study in Nebraska	Cohort	Nebraska, US	Population of Nebraska, 93% of land used for farms or ranches holding 40% of the population (750,000 of 1.8 million)	Incidence rates derived from the Nebraska PD registry between 1997 and 2008	Increased PD incidence associated with higher usage of atrazine, broxomy, alachlor, metribuzin, and glyphosate No association with others pesticides such as paraquat or chlorpyifos	Statewide observation in a large rural area. No relative incidence ratios reported	Wan et al., 2016
Observation in Quebec, Canada	Cohort	Region of Quebec, CAN	290 PD cases of incident PD	detailed questionnaire regarding social, professional, and medical history	Significantly younger age of onset in persons with pesticide exposure (54.75 vs. 59.26 years; *p* = 0.005)	Observation of incident PD cases	Gamache et al. 2019
Taiwan National Health Insurance (NHI) program	Retrospective analysis	Taiwan	9,128 Persons with reported organophosphate or carbamate poisoning between 2000 and 2011 36,446 randomly selected controls	Investigation of the PD incidence ratio in organophosphate/carbamate poisoned and non-poisoned persons	Increased overall PD incidence rate in poisoned persons (adjusted IRR: 1.36; 95% CI 1.26–1.47)	Investigation of cases with acute pesticide poisoning, no investigation of cumulative lifetime exposure	Chuang et al. 2017

In one analyisis of 368 PD cases and 341 controls combined exposure–paraquat and maneb between 1974 and 1999 was associated with increased PD risk (OR 2.14; 95% CI 1.24–3.68). However, exposure restricted to 1990–1999 was not significantly associated (OR 0.93; 95% CI 0.45–1.94). Notably, exposure between 1974 and 1989 was significantly associated with PD risk in both younger (< 60 years; OR 4.17; 95% CI 1.15–15.16) and older (> 60 years; OR 2.15; 95% CI 1.15–4.02) individuals. Over the longer time window (1974–1999), increased risk remained significant only in younger individuals (< 60 years; OR 5.07; 95% CI 1.75 and 14.71), with only a trend observed in older participants (> 60 years; OR 1.36; 95% CI 0.83–2.23). The authors concluded that paraquat and maneb may act synergistically, particularly when exposure occurs early in life (Costello et al. 2009).

Another analysis examined well water concentrations of pesticides in different counties in the study area and found that diazinon, an organophosphate, was linked–a slightly elevated PD risk (OR 1.58; 95% CI 1.03–2.43) (Gatto et al. 2009), though the evidence was rated very low due–the indirect exposure measurement. In another study of 362 PD cases and 341 controls, ziram exposure at both residence and workplace was associated with a significantly increased PD risk (OR 3.01; 95% CI 1.69–5.38), whereas exposure at residence or workplace alone was not. A similar pattern was observed for paraquat (combined exposure: OR 1.5; 95% CI 1.03–2.18). By contrast, maneb exposure at residence (OR 1.71; 95% CI 1.06–2.77), workplace (OR 1.77; 95% CI 1.02–3.09), or both (OR 2.26; 95% CI 1.22–4.20) was consistently associated with increased PD risk (Wang et al. 2011).

Further analysis confirmed these associations. Paraquat exposure was linked–PD risk (OR 1.43; 95% CI 1.43; 95% CI 1.11–1.84) independent of traumatic brain injury, in an analysis that addressed the association between traumatic brain injury and paraquat exposure (Lee et al. 2012). In an analysis of 357 PD cases and 807 controls, household use of organophosphate pesticides was linked to PD (OR 1.71; 95% CI 1.21–2.41), with chlorpyrifos specifically showing a significant association (OR 2.73; 95% CI 1.03–7.24) (Narayan et al. 2013). In another analysis of 357 PD cases and 750 controls, a job exposure matrix–estimate pesticide exposure was used and exposure stratified this in low, medium, and high. Medium (OR 1.61; 95% CI 1.00–2.62) and high exposure (OR 1.97; 95% CI 1.11–3.48), but not low exposure (OR 1.17; 95% CI 0.61–2.24) was linked–increased PD risk (Liew et al. 2014).

In another analysis of 357 PD cases and 752 healthy controls, the authors investigated residence and workplace exposure–different organophosphates and found that workplace only (OR 4.74: 95% CI 2.27–9.92) and both workplace and residential exposure (OR 5.93; 95% CI 1.69–20.87)–demeton was associated with the highest PD risk, whereas residential only exposure to merphos (OR 3.13; 95% CI 1.67–5.79) was associated with the highest PD risk (Wang et al. 2014). Another analysis comapring occupational pesticide use with no occupational use in 360 PD cases and 827 controls found an overall increased PD risk pesticide (OR 2.5; 95% CI 1.5–4.5). Stratification by functional class revealed significant associations for herbicides (OR 2.45; 95% CI 1.37–4.36), insecticides (OR 2.1; 95% CI 1.22–3.60), and fungicides (OR 3.11; 95% CI 1.65–5.88) (Narayan et al. 2017). Among chemical families carbamates were significantly associated with PD (OR 5.55; 95% CI 1.81–17.04), whereas organochlorine and organophosphates showed non-significant trends (Narayan et al. 2017). The levels of evidence from the PEG study ranged from very low–moderate, with large effect sizes and dose-response trends supporting upgrading.

### Case-control study from the Netherlands

A Dutch case-control study recruited 444 PD cases and 876 controls between April 2010 and June 2012 across five hospitals in four regions of the country (van der Mark et al. 2014). Correlations between exposure–different pesticide classes (herbicides, fungicides, insecticides) were strong (*r* = 0.73–0.83), indicating concurrent exposure–multiple pesticides. While there were trends towards increased PD risk associated with high pesticide exposure and individual functional groups, no association was statistically significant and no clear dose-response effect could be observed. High exposure–benomyl was significantly associated with PD risk (OR 2.46; 95% CI 1.16–5.22), but low exposure showed a trend toward decreased risk (OR 06.9; 95% CI 0.29–1.66) (van der Mark et al. 2014). The general level of evidence for low, despite a trend toward dose-response effects. Nevertheless, this study highlights the challenge of disentangling individual pesticide exposures due to correlated usage patterns.

A follow-up analysis estimated exposure based on residential proximity–the place of pesticide use. The strongest association was observed for fenpropimorph (OR 2.79; 95% CI 1.33–5.89). Other pesticides, including paraquat, lindane, maneb, and benomyl, showed non-significant trends toward increased PD risk (Brouwer et al. 2017). Despite the strong association with fenpropimorph evidence quality remained low due–indirect exposure estimation.

### Group health Cooperative, Washington State, USA

The Group Health Cooperative recruited 156 PD cases and 241 controls between 1992 and 2002 (Firestone et al. 2005), later expanding–404 PD cases and 526 controls (Firestone et al. 2010). In one analysis, the authors found no increased PD risk associated with overall occupational pesticide exposure (1.01; 95% CI 0.53–1.92). Herbicide exposure showed a trend towards higher PD risk (OR 1.41; 95% CI 0.51–3.88), whereas insecticide (OR 0.88; 95% CI 0.44–1.76) and fungicide (0.38; 95% CI 0.07–2.05) showed opposite trends. A similar pattern was observed for home-bases pesticide use with no association for overall pesticide use (OR 0.95; 95% CI 0.66–1.37), a trend towards increased risk with herbicides (1.41; 95% CI 0.51–3.88), and opposite trends with insecticides (OR 0.88; 95% CI 0.44–1.76) and fungicides (OR 0.55; 95% CI 0.29–1.05) (Firestone et al. 2005). In the extended study groups, the authors investigated differences in PD risk between men and women and found a trend towards reduced PD risk in men (OR 0.6; 95% CI 0.3–1.29), and a trend towards elevated PD risk in women (OR 3.9, 95% CI 0.39–39.4). However, the number of exposed women was too low–draw conclusions (Firestone et al. 2010). In summary, the Group Health Cooperative did not provide convincing evidence of an association between pesticide exposure and PD risk, resulting in a very low GRADE rating.

### Geoparkinson study

The Georparkinson study was conducted in five regions across Europe. In one publication the authors investigated 767 PD cases and matched them to 1989 controls. Using a job exposure matrix, they estimated exposure to several environmental factors, including pesticides, stratified into low and high exposure. While low exposure was associated with a trend towards higher PD risk (OR 1.09; 95% CI 0.77–1.55), high exposure significantly increased the risk for PD (1.39; 95% CI 1.02–1.89) (Dick et al. 2007). No factors were identified to downgrade or upgrade the level of evidence in this study, resulting in an overall low level of evidence.

### Case-control study from Italy

A case control study conducted in six neurology departments in Italy enrolled 694 PD cases and 640 controls. The authors investigated multiple lifestyle factors, including pesticide exposure, which was associated with a significantly increased PD risk (OR 2.99; 95% CI 1.8–5.1) (Belvisi et al. 2020). Due to the large effects observed and absence of reasons for downgrading, the level of evidence of this study was rated as moderate.

### Case-control study from Pakistan

A large case-control study conducted in Pakistan, matched 600 PD cases–1200 controls. The authors investigated the association between PD risk and the use of several pesticides. While pesticide use in general was associated with a strong increase in PD risk (OR 4.52; 95% CI 2.49–8.19), only the use of aldrin, an organochloride, was individually associated with a significantly increased PD risk (OR 11.0; 95% CI 4.02–30.07). However, the group exposed–aldrin was by far the largest (132 cases and 30 controls), whereas the number of users of other pesticides ranged from only 6–30, leading–very wide confidence intervals (Tufail 2019). Despite the strong effect—the second strongest among all studies - the quality of evidence was rated very low due–a very high risk of bias, as the study was conducted a single hospital. Although the effect of aldrin would qualify for upgrading, the general pesticides did not.

### Case-control study from the united States and Canada

In a case-control study performed in seven regions in the United States and one region in Canada, 519 PD cases were matched–511 controls. The authors assessed use of pesticides in general as well as the use of eight specific pesticides. General use of pesticide was associated with a significantly increased PD risk (OR 1.90; 95% CI 1.12–3.21). Furthermore, the use of 2,4-dichlorophenoxyacetic acid was associated with an elevated PD risk (OR 2.59; 95% CI 1.03–6.48), while no significant associations were found for other pesticides, most likely due–the low number of exposed individuals (0–9 per group) (Tanner et al. 2009). The overall level of evidence of this study was rated low.

### Case-control study from Germany

In a German case control study, PD patients were recruited from nice neurological clinics. A total of 380 patients were matched–controls. Among various environmental factors, pesticide exposure was quantified in “dose-years”, weighted by frequency of use (rarely = 1; for special indications = 2; seasonally = 3). The study found a trend toward increased PD risk associated with herbicide use (OR 2.2; 95% CI 0.9–5.2) and insecticide use (OR 1.6; 95% CI 0.7–3.4) compared–neighborhood controls, and a statistically significant risk associated with herbicide use compared–regional controls (OR 2.4; 95% CI 1.0–6.0). Specifically, exposure to organochlorides, alkylated phosphates and carbamates was associated with elevated PD risk (Seidler et al. 1996). Despite the large effect, the overall quality of evidence was rated low due–wide confidence intervals.

### Case control study from North Carolina

In a family-based case control study conducted in North Carolina between 2000 and 2006, 319 PD cases were matched to 296 controls, of whom 252 were relatives of the cases. Pesticide exposure was assessed by telephone questionnaires. The authors found a significantly increased PD risk associated with a history of direct pesticide application (OR 1.61; 95% CI 1.13–2.29). Higher risk was observed with more frequent exposure (> 10 days/year: OR 2.07; 95% CI 1.26–3.42), longer duration (> 26 years: OR 1.86; 95% CI 1.16–3.00) and greater cumulative exposure (> 215 days (OR 2.37; 95% CI 1.42–3.94). Interestingly, in a sub-analysis stratified by family history of PD, the association between pesticide exposure and PD risk was present only in individuals without a family history of PD, suggesting a stronger impact of pesticides in sporadic PD (Hancock et al. 2008). No factors were identified–downgrade or upgrade the evidence, resulting in a low level of evidence.

### Case-control study from Australia

In a case-control-study conducted in the 1990 s in Australia, 224 PD cases were matched to 310 controls. The authors asked about pesticides/herbicides exposure (used as one variable) and found no significant association with PD risk (OR 1.2; 95% CI 0.8–1.5). They also assessed rural residency, which was associated with increased PD risk (OR 1.8; 95% CI 1.7–2.5), but the association did not remain significant in logistic regression (McCann et al. 1998). The overall level of evidence was rated as very low.

### Case-control study from Hong Kong

A case-control-study conducted in the 1990 s in Hong Kong investigated several PD risk factors in 215 PD cases and 313 controls. There was a trend towards increased PD risk associated with pesticide exposure in farming (OR 1.80; 95% CI 0.903–3.58). I a sex-stratified analysis, women showed a significantly elevated risk (OR 6.84; 95% CI 1.90–24.7), whereas men showed a non-significant trend towards reduced risk (OR 0.68; 95% CI 0.25–1.83) (Chan et al. 1998). These contradictory results combined with wide confidence intervals led–downgrading of the evidence to very low level.

#### Smaller case-control-studies with fewer than 200 cases

The literature search revealed 11 smaller case-control studies with fewer than 200 PD cases investigating the association between pesticide exposure and PD risk. One study conducted in Eastern India reported the highest risk estimate (OR 17.4; 95% CI 4.97–58.84) (Sanyal et al. 2010), but evidence quality was very low. A study from Minnesota, USA, found an increased PD risk only in men (OR 2.4; 95% CI 1.1–5.4) and a non-significant trend towards reduced risk in women (0.6; 95% CI 0.2–1.9) (Frigerio et al. 2006). A study from New York, USA, reported a significantly increased PD risk (OR 2.84; 95% CI 1.34–6.00), though its reliance on self-administered paper surveys introduced high risk of bias (Shermon et al. 2022). A study from the Detroit area found elevated PD risk associated with herbicide (OR 4.1; 95% CI 1.37–12.24) and insecticide exposure (OR 3.55; 95% CI 1.75–7.18), but not with fungicides (OR 1.60; 95% CI 0.47–5.45) (Gorell et al. 1998). In Calgary, Canada, herbicide use was associated with increased PD risk (OR 2.36; 95% CI 1.10–5.04) (Semchuk and Love 1995). A study from Taiwan identified significantly increased PD risks linked to rural residence (OR 2.04; 95% CI 1.23–3.38), farming (OR 1.81; 95% CI 1.25–2.64) and use of herbicides/pesticides (OR 2.89; 95% CI 2.28–3.66). Paraquat use in particular was associated with elevated PD risk (OR 3.22; 95% CI 2.41–4.31) (Liou et al. 1997). A study from Belgrade reported increased PD risk with insecticide exposure (OR 3.22; 95% CI 1.32–7.87) (Vlajinac et al. 2010). In Brazil, direct management of pesticides at workplace (OR 3.78; 95% CI 1.92–7.45) and history of pesticide exposure at workplace (OR 2.35; 95% CI 1.36–4.06) was associated with increased PD risk (Silvestre et al. 2020). A study from southwestern France found significantly increased PD risk (OR 2.20; 95% CI 1.11–4.34) associated with pesticide exposure (Baldi et al. 2003a). A case-control study from east Texas examined multiple pesticides and reported strong associations with rotenone (OR 10.0; 95% CI 2.9–34.3) and chlorpyrifos (OR 2.0; 95% CI 1.02–3.8) use (Dhillon et al. 2008).

**Fig. 1:**
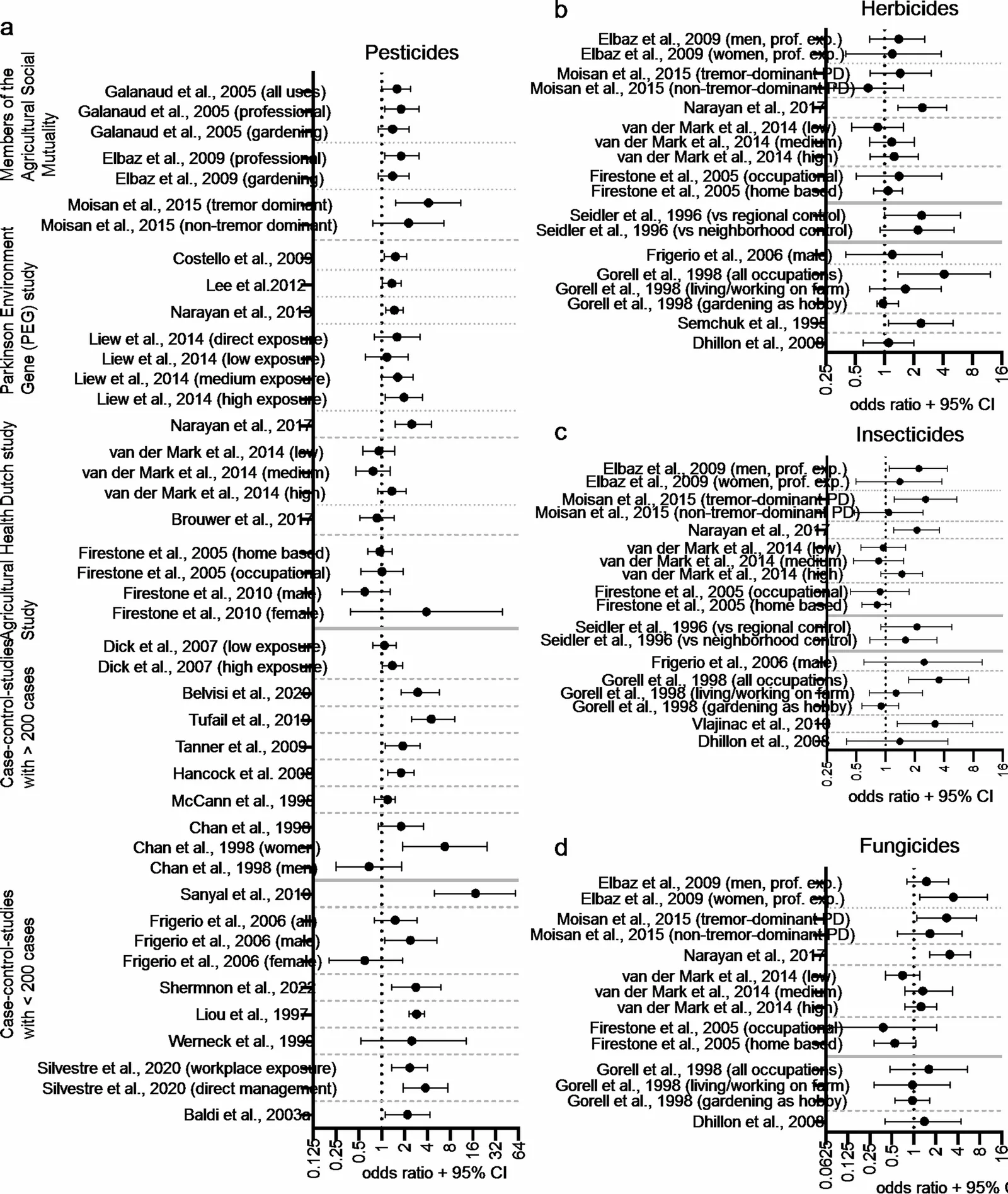
Forest plots showing results of case-control studies. The plots shows the odds ratios with the error bars indicating the 95% confidence intervals. **a** Results of case-control studies that reported an overall effect of pesticide exposure on PD risk. Dotted horizontal lines separate different publications with the same or overlapping study populations. Dashed horizontal lines separate studies within the same category (i.e. studies with more than one publication, larger studies with > 200 cases, smaller studies with fewer < 200 cases). Solid lines separate the different categories. **b** Results from studies that reported effects of herbicides. **c**: Results from studies that reported effects of insecticides. **d** Results from studies that reported effects of insecticides

**Fig. 2:**
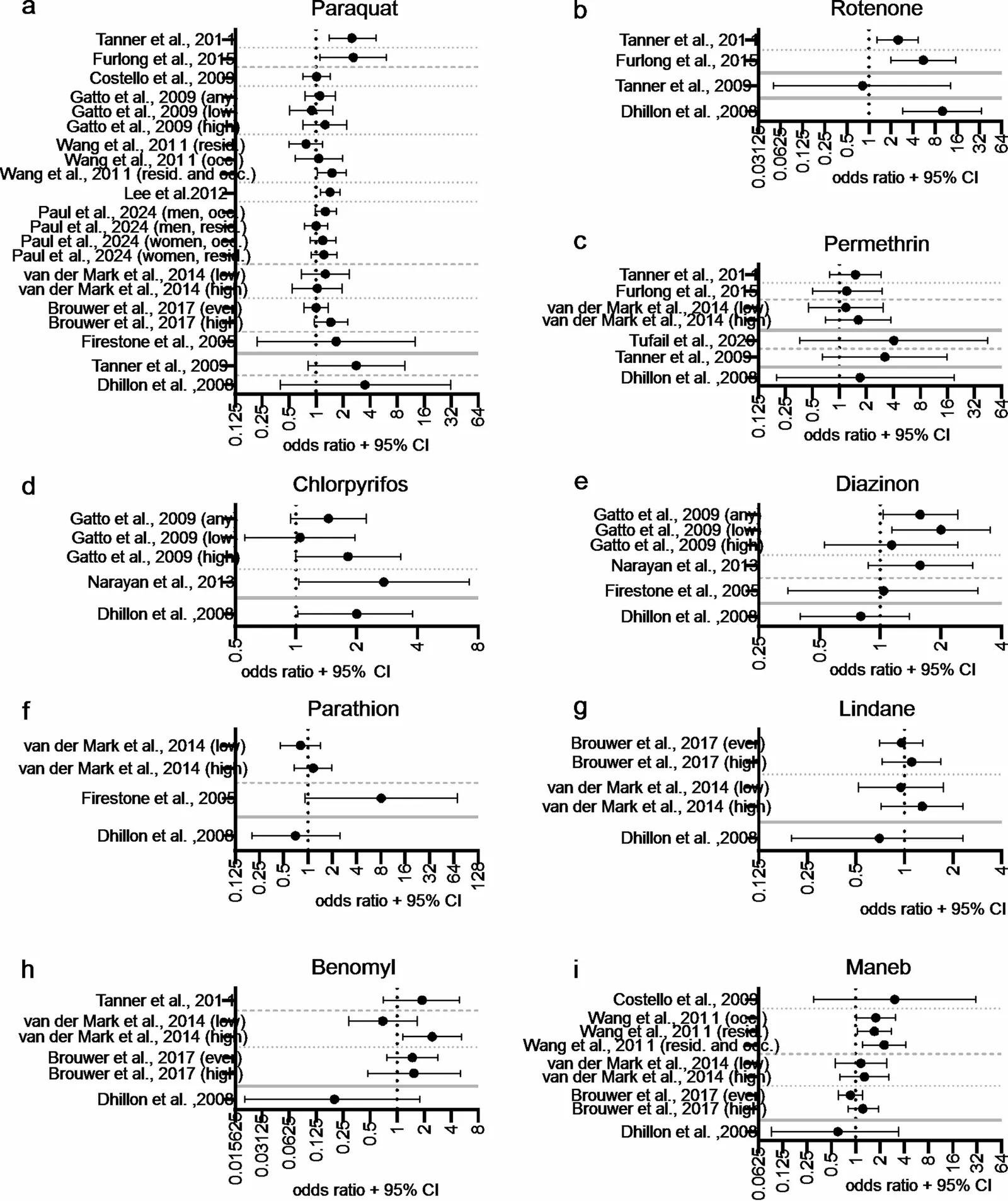
Forest plots showing results of individual pesticide with three or more studies available. The plots shows the odds ratios with the error bars indicating the 95% confidence intervals. For the quaternary ammonium paraquat (**a**), most data were available. Other studies reported on the insecticide rotenone (**b**), the pyrethroid permethrin (**c**), the organophosphates chlorpyrifos (**d**), diazinon (**e**), and parathion (**f**), the organochloride lindane (**g**), the benzimidazole benomyl (**h**), and the dithiocarbamate maneb (**i**)

**Fig. 3:**
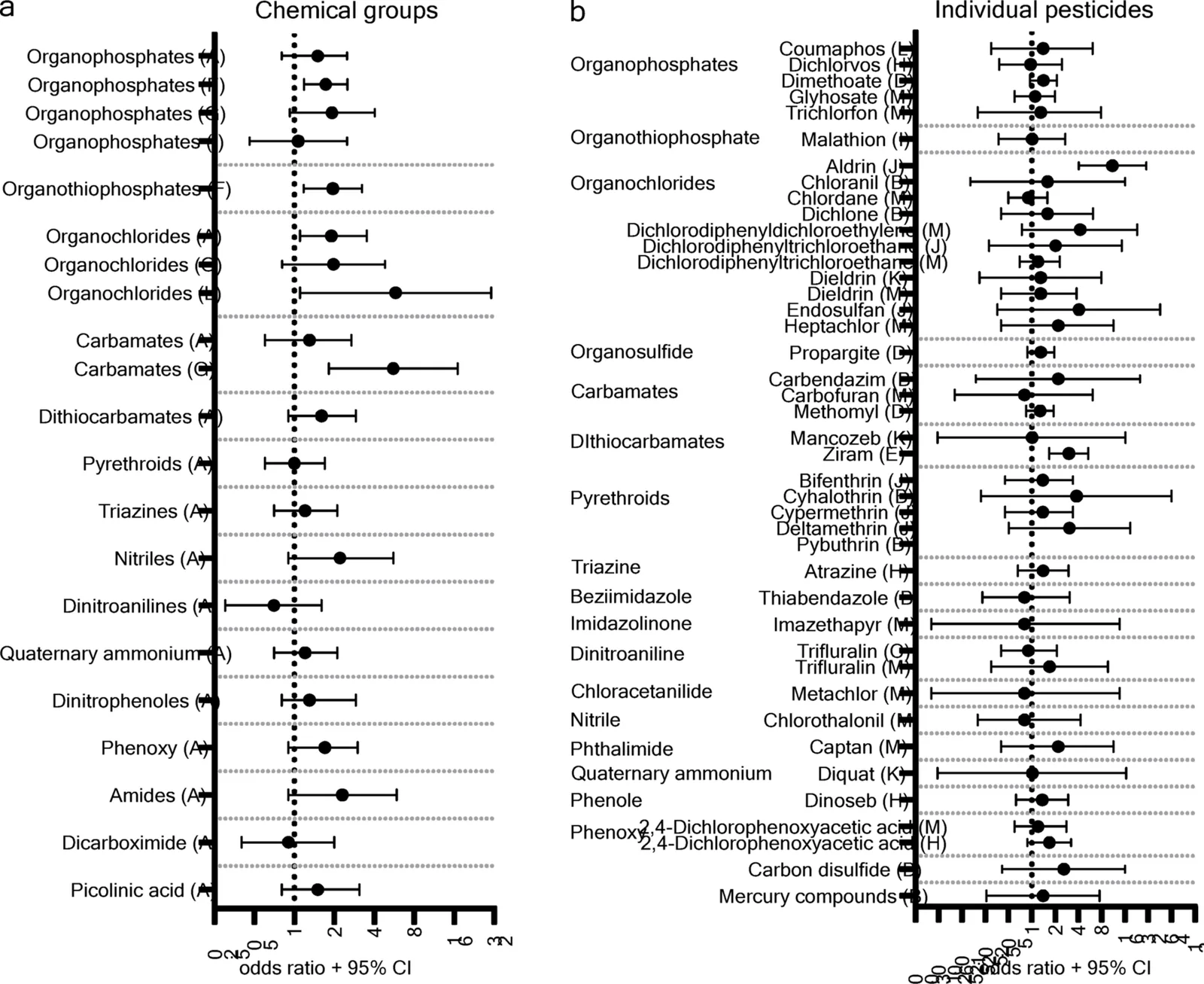
Forest plots showing the results of chemical classes and individual pesticides with less than three studies available. The plots shows the odds ratios with the error bars indicating the 95% confidence intervals. **a** Results of case control-studies for different chemical classes of pesticides. **b** Results of case control-studies for individual pesticides. The letters in brackets indicate the publication. The overall order is the same as in Table 1. Elbaz et al. (2009); B: Tanner et al. (2011); C: Furlong et al. (2015); D: Gatto et al. (2009); E: Wang et al. (2011); F: Narayan et al. (2013); G: Narayan et al. (2017); H: van der Mark et al. (2014); I: Firestone et al. (2005); J: Tufail et al. (2020); K: Tanner et al. (2009): L: Seidler et al. (1996); M: Dhillon et al. (2008)

 The available data on PD risk in association with pesticide exposure from case-control studies are illustrated in Fig. 1a with a distinction by functional groups illustrated in Fig. 1b–d. Figures 2 and 3 show the data from studies that reported an association between PD risk and individual pesticides.

Overall, most case-control studies demonstrated trends or significant associations between pesticide exposure and increased PD risk. However, the overall quality of evidence was low to moderate. Only a few studies (Dick et al. 2007; Liew et al. 2014; van der Mark et al. 2014) were designed to assess dose-response effects. Furthermore, only some studies distinguished between functional groups of pesticides (Fig. 1b–d), and even fewer analyzed individual pesticides (Figs. 2 and 3). Results were not always consistent: for example, some studies reported higher risks in women (Firestone et al. 2010; Chan et al. 1998), whereas others suggested the opposite (Frigerio et al. 2006). Subgroup analyses were often limited by small case numbers, reducing data quality and leading to wide confidence intervals.

### Cohort studies and registries

The following section presents cohort and registry studies. Because of their purely observational nature and the heterogeneity of study design, a GRADE rating was not performed. The results of these studies are presented in Table 2.

### PAQUID study in France

In prospective cohort study from France, which enrolled participants from 1987 to 1998, the authors investigated individuals aged over 65 years at inclusion. In total, 1507 participants were enrolled, of whom 320 reported pesticide exposure. During follow-up, a significant association was observed between PD incidence and occupational pesticide exposure, but only in men (OR 5.63; 95% CI 1.47–21.58), with no significant association in women (OR 1.02; 95% CI 0.11–4.82). Interestingly, more women (922) than men (585) were included in this study (Baldi et al. 2003b).

### French agricultural cohort AGRICAN, Mutualité sociale agricole (MSA)

The AGICAN cohort enrolled all active and retired agricultural workers between 2005 and 2007. In the French health system, all agricultural workers are affiliated with the Mutualité Sociale Agricole (MSA), the agicultural health insurance provider. In AGRICAN, data of 1,728 self-reported PD cases and 148,078 healthy controls were analyzed. Pesticide exposure was assessed using the crop-exposure-matrix PESTIMAT (Pouchieu et al. 2018), which is based on annual reports about crops and active pesticide agents (Baldi et al. 2017). The authors reported increased PD risk among individuals with a history of pesticide poisoning (OR 1.51; 95% CI 1.14–1.99 for once poisoning; OR 2.25; 95% CI 1.52–3.38 for multiple poisonings). They also found significantly elevated risks associated with exposure to several dithiocarbamates (ORs 1.31–1.52; 95% CI from 1.08–1.60–1.26–1.83), rotenone (OR 1.57; 95% CI 1.08–2.29), paraquat (OR 1.43; 95% CI 1.17–1.75), and diquat (OR 1.39; 95% CI 1.14–1,68) (Pouchieu et al. 2018).

Another French study used the French National Health Insurance database–identify individuals reimbursed for antiparkinsonian drugs between 2009 and 2015. Newly diagnosed PD cases were identified via algorithm and filtered by MSA affiliation. In total, 10,282 PD cases were analyzed, and pesticide expenditures were stratified by farming type. A significantly increased PD incidence was found in vineyards areas with high pesticide expenditures (incidence rate ratio (IRR): 1.16, 95% CI 1.06–1.28), while no significant effect was observed for other types of farming (Perrin et al. 2021). This study is notable, because the French health system allowed a near-complete coverage of the French population during the observation period.

### The TRACTOR project in France

The largest French epidemiological study, the TRACTOR project, analysed insurance data from 1,088,561 farm managers, betweeb 2002 and 2016. Among these, 8,845 developed PD (Petit et al. 2025). Harzard ratios (HRs) for PD incidence were calculated across different agricultural activities. Arboriculture, pig farming, dairy farming, mixed cattle farming, and crop farming were associated with significantly elevated HRs (> 1.3). Other activities, including cow farming, truck farming, and mixed or unspecific farming, showed moderately elevated HRs (> 1, < 1.2). Trends towards increased HRs were alss observed for viticulture and ovine/caprine farming. By contrast, gardening, landscaping, and reforestation, and small animal farming, were associated with reduced HRs (Petit et al. 2025).

### Italian longitudinal study on aging (ILSA)

The ILSA investigated randomly selected individuals aged 65–84 years across eight Italian regions in March 1992. Of 44,737 individuals, approximately 5,000 were screened for PD, and 113 PD cases were identified. The authors examined several risk factors and reported increased PD risk among persons holding a pesticide-use license (OR 3.68; 95% CI 1.57–8.64). However, only 7 PD cases and 83 controls possessed such a license, limiting meaningfulness (Baldereschi et al. 2003).

### Netherlands cohort study on diet and cancer

The Netherlands Cohort Study on diet and cancer enrolled 120,852 individuals aged 55–69 years in 1986. In a follow-up analysis, PD-related deaths were assessed up to 2003. Pesticide exposure was estimated using a job-exposure-matrix and stratified into low and high exposure and cumulative exposure. In total, 402 PD-related deaths occurred in men and 207 in women. Trends towards increased PD mortality were observed (low exposure: HR 1.35; 95% CI 0.81–2.26), high exposure: HR 1.27; 95% CI 0.86–1.88). Significantly increased PD mortality was found in the group with the shortest cumulative exposure 1 to 27 unit years; HR: 1.89; 95% CI 1.11–3.22), whereas longer exposure durations did not yield a significant associations (Brouwer et al. 2015). This is particularly interesting, because the case numbers in the different exposure groups were comparable.

### The agricultural health study (AHS)

The AHS, initiated in the 1990 s in Iowa and North Carolina, USA, is a prospective observational study of pesticide users and their spouses (Alavanja et al. 1996). Exposure was assessed by questionnaires. Both prevalent PD at baseline (1993–1997) and incident PD during follow-up (1999–2003) were analyzed (Kamel et al. 2007). Interestingly, the study observed inverse trends between pesticide use and PD prevalence. Among never pesticide use, 15 PD cases occurred among 13,837 individuals, whereas 67 cases occurred among 65,183 pesticide users (OR 0.5; 95% CI 0.2–1.1). Similar non-significant trends were observed with pesticide mixing and application. When cumulative lifetime days of exposure were assessed, higher exposure groups showed no increased prevalence (65–200 days: 0.7; 95% CI 0.3–1.3; 201–396 days: 0.7; 95% CI 0.4–1.4; ≥ 397 days: 0.8; 95% CI 0.4–1.5) compared–individuals with 0–64 days of pesticide exposure, but PD incidence was elevated among those with ≥ 397 exposure days (OR 2.3; 95% CI 1.2–4.5). PD incidence was also linked–specific pesticide-related tasks (mixing pesticides > 50% of the time: OR 1.2; 95% CI 0.5–2.7; pesticide application: <50% of the time: OR 1.2; 95% CI 0.5–3.1; >50% of the time; OR 1.9; 95% CI 0.7–4.7). Compound-specific analyses revealed increased PD prevalence with several herbicides, including paraquat, a quaternary ammonium compound (OR 1.8; 95% CI 1.0–3.4), and cyanazine, a triazine (OR 2.6; 95% CI 1.4–4.9) and a trend (OR ≥ 1.4) for pendimethalin, a dinitroaniline (OR 1.4; 95% CI 0.8–2.6). Regarding PD incidence, increased risks were found for trifuralin, a dinitroaniline (OR 1.7; 95% CI 1.0–3.2), and 2,4,5-trichlorophenoxyacetic acid (OR 1.8; 95% CI 1.0–3.3) and a trend for butylate, a thiocarbamate (OR 1.4; 0.8–2.5). In the group of insecticides, trends towards a higher PD incidence were observed with exposure–lindane, an organochloride (OR 1.4; 95% CI 0.8–2.5), and phorate, an organothiophosphate (OR 1.4; 95% CI 0.8–2.5). Among fungicides, similar trends were observed with chlorothalonil, an aromatic compound (OR 2.0; 95% CI 0.9–4.4), and benomyl, a benzimidazole (OR 1.7; 95% CI 0.7–3.7). However, none of the insecticides or fungicides were associated with a statistically significant increased PD prevalence or incidence (Kamel et al. 2007). In a follow-up analysis, 66,110 individuals were included, of which 491 were diagnosed with PD, 373 of them pesticide applicators and 118 spouses. The analysis showed that ever-use of terbufos, a phosphoric acid ester insecticide (HR 1.31, 95%CI1.02–1.68), trifluralin, an organofluorine herbicide (HR 1.29, 95%CI 0.99–1.70), and 2,4,5-trichlorophenoxyacetic acid, a chlorophenoxy acetic acid herbicide (HR:1.57, 95%CI1.21–2.04) increased PD risk (Shrestha et al. 2020).

### Registry studies from Washington state

One cohort from the Washington State of Health recalled participants originally enrolled between 1972 and 1976. Using self-administered questionnaires, participants reported farm employment, pesticide exposure, and well-water use. In total, 238 persons reported pesticide exposure and 72 did not. No association were observed between PD prevalence and well-water use, farm employment or exposure–individual pesticides. However, PD prevalence (adjusted PR: 2.0 95% CI 1.0–4.2) was increased among those with the longest pesticide exposure (Engel et al. 2001).

Another study from Washington State analyzed PD-related deaths between 2011 and 2015. Premature death was defined as occurring before age of 75. Among 4,591 PD-related deaths identified, 659 met the criteria for premature death. Exposure estimates, derived from a crop-exposure matrix, incorporating data for residential addresses, well locations, and occupational data. Premature mortality was significantly associated with glyphosate exposure (OR 1.33; 95% CI 1.06–1.67), with a positive trend for paraquat (OR 1.22; 95% CI 0.99–1.51) (Caballero et al. 2018).

### California vital statistics

A California study also investigated PD-related mortality using pesticide-use reports from 1972–1990–stratify counties in no, low, moderate, and high pesticide-use. Increased prevalence odds ratios (POR) for PD as underlying cause of death was found in counties with high pesticide use (death from 1989 to 1994: POR 1,45; 95% CI 1,32–1,59, from 1984 to 1988: POR 1.19; 95% CI 1.06–1.34) (Ritz and Yu 2000).

#### Cancer prevention study (CPS) II nutrition cohort

The CPS II Nutrition Cohort, initiated in 1992, includes over 180,000 participants. Originally designed–investigate cancer risk factors, the 2001 follow-up also assessed PD. Among > 140,000 respondants, 7,864 reported pesticide exposure, while 135,461 did not. A total of 370 PD cases were identified, including 43 with pesticide exposure. Pesticide exposure was associated with increased PD risk (risk ratio [RR]: 1.8 (95% CI 1.3–2.5), regardless of sex, age (< 65 years; ≥ 65 years), or smoking status. Specific pesticide date were not available (Ascherio et al. 2006).

### Colorado, USA

Using a registry of 332,971 Medicare beneficiaries, the authors identified 4,207 PD cases. Pesticide concentrations in groundwater were correlated with PD prevalence. A small, yet significant, association was observed between overall pesticide concentrations and PD prevalence (OR 1.03; 95% CI 1.02–1.04). Atrazine levels were particularly associated (OR 1.04; 95% CI 1.03–1.05). The authors estimated a 3% increased PD risk per 0.01 mg/L pesticide and a 4% increase per 0.01 mg/L atrazine in groundwater (James and Hall 2015). Unlike most studies, exposure here was based on measured environmental concentrations.

### Cohort study in Nebraska

A Nebraska study, conducted in a state where 93% of the land is used for agriculture and 40% of residents live in rural areas, examined associations between pesticide use and PD incidence. Common pesticides included glyphosate, atrazine, acetochlor; 2,4-dichlorophenoxyacetic acid, and metolachlor. Increased PD incidence was observed with higher use of atrazine, broxomy, alachlor, metribuzin, and glyphosate, but not with other pesticides, such as paraquat or chlorpyifos. Interestingly, no association was found between the proportion of farmers in a region and PD incidence, suggesting that non-occupational pesticide exposure may play a stronger role than occupational exposure (Wan and Lin 2016).

### Quebec, Canada

In a study 290 incident PD cases from Quebec, individuals reporting pesticide exposure had a significantly younger age of onset (54.75 vs. 59.26 years; *p* = 0.005) compared with non-exposed individuals (Gamache et al. 2019).

### Taiwan

A Taiwanese study investigated individuals reporting acute organophosphate or carbamate poisoning between 2000 and 2011. Among 9128 poisoned individuals, PD incidence was higher (adjusted IRR: 1.36; 95% CI 1.26–1.47) than among 36,446 randomly selected controls (Chuang et al. 2017).

Overall, the data from the above studies support a trend toward increased PD risk associated with pesticide exposure.

### Meta-analysis

A 2023 meta-analysis examined studies specifically investigating the association between glyphosate exposure and neurological conditions. In summary, the authors concluded that there was no convincing evidence linking glyphosate exposure–adverse neurological outcomes (Chang et al. 2023).

### Gene pesticide interaction

A study in the PEG cohort investigated interactions between genetic variants and pesticide exposure in relation–Parkinson’s disease (PD) risk Variants in the gene encoding the human dopamine transporter (*DAT/SLC6A3*) were associated with increased susceptibility–the pesticides maneb and paraquat. Additionally, variants in ABCB1 (ATP Binding Cassette Subfamily B Member 1), which encodes the efflux transporter P-glycoprotein (also known as Multidrug Resistance Protein 1), appeared–increase vulnerability–organochlorines and organophosphates Additionally, variants in *ABCB1* (ATP Binding Cassette Subfamily B Member 1), which encodes the efflux transporter P-glycoprotein (also known as Multidrug Resistance Protein 1), appeared–increase vulnerability–organochlorines and organophosphates. Supporting evidence also indicated that variants in *PON1*, which encodes the detoxifying enzyme paraoxonase, result in slower metabolism of organophosphates such as diazinon, chlorpyrifos, and parathion, potentially contributing–PD risk. Moreover, variants in *NOS1*, encoding nitric oxide synthase, may amplify oxidative stress in response–organophosphate exposure. A haplotype in *ALDH2*, encoding aldehyde dehydrogenase, was also associated with increased PD risk following pesticide exposure (Ritz et al. 2016). However, a genome-wide gene–environment interaction analysis found no significant associations between PD risk and genetic variants at the genome-wide level (Biernacka et al. 2016).

### Algorithms–quantify pesticide exposure

One of the main challenges in evaluating epidemiological studies on the association between pesticide exposure and the risk of developing PD is accurately quantifying pesticide exposure. Most studies rely on questionnaires or interviews to collect data on pesticide use (Galanaud et al. 2005; Elbaz et al. 2009; van der Mark et al. 2014; Furlong et al. 2015; Moisan et al. 2015; Hancock et al. 2008; Seidler et al. 1996).

In the AHS, a detailed algorithm was developed–improve exposure assessment. This scoring system incorporates several factors, including the method of pesticide application, use of personal protective equipment (PPE), and how the PPE was cleaned. Additionally, it accounts for the duration and frequency of exposure (Dosemeci et al. 2002).

Another widely used approach is the Job Exposure Matrix (JEM) (Matheson et al. 2005; van der Mark et al. 2014). JEMs estimate exposure by assigning weights–specific tasks, such as pesticide mixing or application, as well as the number of years worked and the intensity of exposure (Liew et al. 2014; van der Mark et al. 2014).

Furthermore, in France, a crop-exposure matrix called PESTIMAT was designed that used regional lists of crops and pesticide use per year and parameters such as probability, intensity, and frequency of use–estimate exposure (Baldi et al. 2017).

### Experimental data

There are only a few case-control studies that presented data on individual pesticides (Fig. 3). Nevertheless, in the following part, experimental data of investigations of the toxicity of various pesticides on dopaminergic neurons or the dopaminergic system using both in vitro and in vivo models of PD are presented. The following section provides an overview of key findings, with a focus on studies that demonstrated significant effects.

### Experimental data on herbicides

Among herbicides, paraquat and the triazine compound atrazine are the most extensively studied in experimental models of PD. Data from case-control studies that reported effects of herbicides as a functional group are heterogeneous, with only a few studies demonstrating a significantly increased risk for PD in association with herbicides (Fig. 1b).

### Paraquat

Multiple studies have investigated the effect of paraquat on the dopaminergic system using in vivo models. One study reported a loss of tyrosine hydroxylase (TH)-positive neurons and increased alpha-synuclein levels in the striatum of mice treated with 10 mg/kg paraquat twice weekly for four weeks (Su and Niu 2015). Other researchers assessed locomotor activity in mice following repeated intraperitoneal injections of 10 mg/kg paraquat, 30 mg/kg maneb, or a combination over six weeks. Only the combined treatment resulted in reduced locomotor activity, with the effect being more pronounced immediately after the injections than 24 h later. Similarly, reduced TH immunoreactivity in the substantia nigra was observed only after the combined treatment, while paraquat or maneb alone had no significant effect (Thiruchelvam et al. 2000). In a subsequent study, the same group demonstrated that treatment with paraquat alone led–reduced locomotor activity in 5- and 18 months old mice, particularly 10 days after the final treatment, although the effect was less pronounced after three months. In contrast, a combined treatment with paraquat and maneb resulted in sustained locomotor deficits persisting for three months after the treatment. Additionally, paraquat alone led to a reduction in striatal TH levels three months after the treatment, whereas no such reduction was observed two weeks post-treatment. The combination of paraquat and maneb, however, causes more substantial reduction in TH levels, already detectable two week after treatment. Maneb alone again showed no significant effects (Thiruchelvam et al. 2003). In line with the first observation, also others found that weekly intraperitoneal injections of 10 mg/kg paraquat for three weeks led–dopaminergic neuron loss in the substantia nigra of mice (McCormack et al. 2005). Others demonstrated that paraquat accelerated alpha-synuclein aggregation in a cell free system in concetrations ranging from 10 to 1000 µM (Manning-Bog et al. 2002).

Paraquat is the pesticide, of which individual effects were reported most frequently in case-control studies. Even though the majority of studies reported trends towards increased PD risk associated with paraquat, the effects were generally heterogenous with some studies even reported negative trends and only a few were able–demonstrate a statistically significant effect (Fig. 2a). In line with that, also experimental studies could not unequivocally show toxic effects of paraquat, with some observing effects only in combination with maneb.

## Triazines

Within the triazine class of herbicides, atrazine is the most extensively studied compound in experimental PD models.

One study demonstrated that oral administration of atrazine for 14 days led to a reduction of dopaminergic neurons in the substantia nigra. Notably, neuronal loss was observed one day after the treatment (day 15) only at the highest dose (250 mg/kg bodywight), while milder cell loss was also detected at lower doses (25 mg/kg and 125 mg/kg) on day 22 and 64. Despite these findings, no differences in TH protein levels were found between treated and control animals on days 15 and 22. Striatal dopamine levels were reduced on day 15 but returned–baseline levels by days 22 and 64 (Coban and Filipov 2007).

In another study, the investigators found that chronic exposure–atrazine (300 mg/kg mixed into food and water) for one year resulted in a reduction in the number of dopaminergic neurons in the substantia nigra and impaired locomotor activity in rats (Bardullas et al. 2013). Similarly, others reported decreased TH levels in both the substantia nigra and striatum of rats treated with 50 mg/kg atrazine for 45 days (Li et al. 2020).

Only one case-control study reported an effect of atrazine, which was not statistically significant (van der Mark et al. 2014) and another study reported an effect of triazines in general, which was also not statistically significant (Elbaz et al. 2009). While experimental data suggest acute toxicity of triazines–dopaminergic cells, epidemiological data are scarce (Fig. 3a, b).

### Organophosphates

#### Glyphosate

Most organophosphates are used as insecticides (see below). However, glyphosate, though structurally an organophosphate, is one of the most widely used herbicides. One group demonstrated that exposure of C. elegans to glyphosate reduced the number of dopaminergic neurons. In an acute exposure paradigm (10% glyphosate for 30 min followed by washing), and a chronic exposure paradigm (9.8% glyphosate for 30 min without washing followed by 24 h of incubation), both resulted in dopaminergic neuron loss. The authors estimated that the chronic exposure model corresponded–approximately 10.5 years of human exposure (Negga et al. 2012). Another study investigated the effects of glyphosate in a mouse model. Mice were intraperitoneally injected with either MPTP or saline and exposed–drinking water containing 0.098% of a glyphosate-based pesticide (with 48% w/v glyphosate) or normal water for three weeks. Glyphosate exposure had no effect on dopaminergic neuron numbers in saline-treated mice. However, in MPTP-treated mice, glyphosate co-exposure led to a greater reduction in dopaminergic neurons in the substantia nigra compared–MPTP treatment alone (Pu et al. 2020).

Only one case-control study reported data on glyphophosate (Fig. 3b). Overall, also a very small and not significant trend for increased PD risk was reported (Dhillon et al. 2008). Furthermore, a recent meta-analysis concluded that no convincing evidence of neurotoxicity of glyphosate was available (Chuang et al. 2017).

### Dithiocarbamates

Most dithiocarbamates are primarily used as fungicides. Accordingly, the relevant data are presented in the section on fungicides.

#### Experimental findings with insecticides

Various insecticides have been evaluations in both in vitro and in vivo PD models.

#### Rotenone

Rotenone, a well-known inhibitor of complex I of the mitochondrial respiratory chain, is the most studied insecticide in this context. However, the results obtained with rotenone are inconsistent. Some studies reported selective dopaminergic neuron loss in the substantia nigra, without affecting the striatum, following rotenone treatment in rats. These finding were accompanied by alpha-synuclein pathology (Sherer et al. 2003). In contrast, other researchers observed neuronal loss in both the substantia nigra and the striatum in rats, along with tau pathology (Höglinger et al. 2005). Still, some studies failed–detect any nigral cell loss after rotenone treatment in rats (Richter et al. 2007). Despite these discrepancies, rotenone has been one of the most widely used compounds in past years for modelling PD in vivo (Ibarra-Gutiérrez et al. 2023).

With the exception of one study, the few case-control studies that reported an effect of rotenone all suggested a significantly increased PD risk associated with rotenone exposure (Fig. 2b). Therefore, in light of the experimental data, rotenone is the pesticide, of which the evidence as a possible cause of PD is strongest.

#### Pyrethroids

Various in vitro and in vivo studies have investigated the effect of pyrethroids, particularly permethrin and fenpropathrine.

In one study, rats were treated with 34 mg/kg body weight of permethrin from postnatal day 6–21. By day 60, the authors observed reduced dopamine levels and decreased number of dopaminergic neurons (Nasuti et al. 2017). In another study, 6.1 mg/kg brain weight of fenpropathrin was injected directly into the striatum of mice. Twenty-four weeks later, the mice exibited a reduced number of dopaminergic neurons in the substantia nigra and decreased TH levels in the midbrain. These neuropathological changes were accompanied by impaired locomotion in both the open field and the rotarod test (Jiao et al. 2020). The neurotoxic effect of pyrethroids are believed–by mediated by their common metabolite 3-phenoxybenzoic acid. Supporting this, it was shown that treatment with both fenpropathrin and 3-phenoxybenzoic acid reduced the viability of SH-SY5Y cells and lowered TH levels in the substantia nigra of mice. Notably, 3-phenoxybenzoic acid induced more pronounced motor deficits than fenpropathrin (Wan et al. 2022). However, not all studies observe similar effects. Some researchers found no reduction in TH levels in the striatum of mice treated once with 200 mg/kg body weight of permethrin (Kou et al. 2006; Dodd and Klein 2009).

Permethrin is one of the pesticides of which individual data from case-control studies as well as experimental data are available. However, even though the data available from case-control studies all showed trends towards increased PD risk no study could demonstrate significant effects, mainly caused by low case numbers resulting in large confidence intervals (Fig. 2c). In addition, also case-control studies that reported about the association between pyrethroids as group (Fig. 3a) or of other pyrethroids (Fig. 3b) could not demonstrate significant effects. Furthermore, not all experimental studies showed a clearly toxic effect of permethrin.

### Organophosphates

Among organophosphate insecticides, in vitro and in vivo studies have investigated the potential neurotoxic effects of dichlorvos, chlorpyrifos, monocrotophos, and parathion. In one study, oral administration of dichlorvos (2.5 mg/kg body weight per day) resulted in a reduction of dopaminergic neurons and alpha-synuclein deposits in the substantia nigra in rats (Binukumar et al. 2010). Another study reported that treatment with 6 mg/kg body weight per day of dichlorvos reduced mitochondrial respiratory chain activity and induced apoptotic cell death in rat brains. However, no specific brain region was reported (Kaur et al. 2007). Oral exposure–monocrotophos led–decreased TH levels in the striatum of mice (Ali and Rajini 2016), while in vitro treatment if PC12 cells with monocrotophos induced apoptosis (Kashyap et al. 2010). Neonatal exposure–5 mg/kg of chlorpyrifos resulted in a reduction of dopaminergic neurons of rats (Zhang et al. 2015). However, other studies did not observe significant effects on striatal TH levels following chlorpyrifos treatment (Kou et al. 2006; Dodd and Klein 2009). Nevertheless, one study found that chlorpyrifos treatment (3 mg/kg bodyweight) exacerbated loss of TH-positive neurons and further increased alpha-synuclein levels induced by treatment with paraquat (Su and Niu 2015). Regarding parathion, in vitro studies have demonstrated detrimental effects of methyl-parathion on SH-SY5Y cells at concentrations above 50 µM (Bharate et al. 2010) and at similar concentrations for ethyl-parathion (Amar et al. 2023). The few case-control studies that reported effects of individual pesticides or chemical groups reported significantly increased PD risk associated with chlorpyrifos exposure, particularly with high exposure (Fig. 2d). Even though, epidemiological data suggest an effect of chlorpyrifos on PD risk, experimental could not convincingly show toxicity of chlorpyrifos for dopaminergic cells. The association with PD risk for other organophosphates was ambiguous (Figs. 2e and f and 3a, b).

### Organochlorines

Organochlorides are commonly used as insecticides. Among them, most experimental data are available for dieldrien. However, chlorinated pesticide, such as lindane, heptachlor, or endosulfan, have also been investigated.

One study showed that treatment with 10 µM dieldrin for 48 h and 100 µM dieldrin for 24 h reduced the number of TH-positive neurons in primary midbrain cultures of fetal rats (Sanchez-Ramos et al. 1998). Others showed that once treatment with 1 and 3 mg/kg bodyweight dieldrin led–increased oxidative stress and increased alpha-synuclein levels in the striatum of rats, however dieldrin treatment did not result in reduced numbers of total neurons or dopaminergic neurons or changes in TH levels (Hatcher et al. 2007). One study demonstrated that intraperitoneal injection of 0.3 mg/kg body weight of dieldrin into female mice every 3 days for 13 weeks, beginning 4 weeks before mating, resulted in reduced motor activity in the male, but not the female, offspring. Although there was no observed loss of dopaminergic neurons, increased dopamine turnover was reported in the male offspring (Gezer et al. 2020). Dieldrin has also been shown to inhibit proteasome activity and induced alpha-synuclein aggregation in alpha-synuclein overexpressing N27 cells, a rat mesencephalic cell line (Sun et al. 2005). Another study found a modest proteasome inhibition (− 4.9%) in SK-MC cells treated with 10 µM dieldrin, and a similar effect (− 3.8%) with 10 µM endosulfan (Wang et al. 2006). Furthermore, endosulfan also exhibited cytotoxic effects in SH-SY5Y cells, but only at concentrations above 100 µM (Jia and Misra 2007). Treatment with 10 µM dieldrin and 50 µM lindane resulted in oxidative stress and reduced viability of N27 cells (Sharma et al. 2010). Additionally, intraperitoneal injection of 7 mg/kg body weight of heptachlor caused dopaminergic neuron loss in mice (Hong et al. 2014). Of the few case-control studies that reported effects of individual organochlorides, only one study from Pakistan(Tufail 2019) found significant associations between organochlorides in general and aldrin in particular and PD risk (Fig. 3a, b), while other studies could not find significant effects with organochlorides (lindane: Figs. 2g; 3a, b). In light of the quite high doses needed–exert toxicity on dopaminergic cells in experimental models and the weak basis of epidemiological data, there is no convincing evidence of an effect of organochlorides on PD risk.

## Experimental findings with fungicides

Among fungicides, the benzimidazole (often referred–as BMC fungicides) benomyl and the dithiocarbamate maneb have been the most extensively studied in experimental models of PD.

### Benzimidazoles

Benomyl is the most thoroughly investigated in relation–dopaminergic toxicity. One study demonstrated that benomyl treatment of mesencephalic primary cultures from postnatal day 1 rats resulted in a dose-dependent reduction of dopaminergic neurons at concentrations of 0.1 µM and 1 µM. In the same study, zebrafish embryos exposed 1 µM benomyl exibited a reduction of VMAT2 positive neurons across several brain regions (Fitzmaurice et al. 2013). The proposed mechanism of action involves inhibition of the aldehyde-dehydrogenase (ALDH), an enzyme critical for dopamine metabolism. ALDH catalyzes the conversion of the toxic intermediate (3,4-dihydroxyphenylacetldehyde (DOPAL)–the less harmful metabolite DOPAC. ALDH inhibition by benomyl may therefore lead–DOPAL accumulation, contributing–toxicity (Fitzmaurice et al. 2013). Supporting this, another study found that benomyl treatment of PC12 cells, a rat pheochromocytoma cell line, reduced the DOPAC/(DOPAL + DOPET) ratio, consistent with ALDH inhibition as mechanism of action of benomyl (Casida et al. 2014).

The data from case-control studies that are available are ambiguous with some studies even showing negative trends for PD risk associated with benomyl exposure (Fig. 2h). After all, the data basis for an effect of benzimidazoles on PD risk is weak.

### Dithiocarbamates

Amongs the dithiocarbamates, maneb is the most extensively studied compound in relation to dopaminergic toxicity. One study reported that daily intrathecal administration of maneb for 14 days reduced dopamine levels in rats (Zhang et al. 2003). Other studies have examined the combined effect of maneb and paraquat. In one study, repeated co-administration of paraquat and maneb resulted in reduced striatal TH levels and reduced TH immunoreactivity and cell count in the substantia nigra of mice, whereas application of paraquat or maneb alone had no significant effect. Furthermore, behavioral tests revealed a transient reduction of locomotor activity following repeated treatment with maneb alone. However, this effect was observed only at the beginning of the behavioral experiment, which was conducted immediately after the 12th injection. After 45 min no difference in activity was detected between maneb-treated and control mice. Notably, co-administration of maneb and paraquat reduced locomotor activity when assessed directly after the 4th or 12th injection. However, behavioral tests conducted 24 h after the injections showed no significant reduction in locomotor activity. Specifically, 24 h after the 4th injection, no significant effect was detectable in mice treated with maneb, paraquat or their combination. In contrast, 24 h after the 12th injection, a transient reduction in locomotor activity was observed, but only in the co-administration group. However, in none of the experimental group did behavioural tests performed 24 h after the injections reveal effects that persisted until the end of the 45-minute sessions. In the same study, a subgroup of mice received MPTP-injections. In this group, paraquat or maneb led–a comparable reduction in locomotor following the MPTP challenge, while co-administration of both compounds exacerbated the effect. Notably, in saline-injected mice, neither maneb nor paraquat alone, nor their co-administration, had significant impact on locomotor activity in this experiment (Thiruchelvam et al. 2000). In a subsequent study by the same group, maneb alone again did not affect behaviour or TH levels, although, a synergistic detrimental effect with paraquat was again observed (Thiruchelvam et al. 2003). Notably, in another study where paraquat alone caused cell loss of TH-positive neurons and increased alpha-synuclein levels (see above), additional treatment with maneb did not exacerbate these effects. In fact, alpha-synuclein levels in the co-treatment were not significantly different from the controls, suggesting that maneb may have attenuated the effect of paraquat on alpha-synuclein levels (Su and Niu 2015). However, in both studies, the effects of maneb alone were not independently assessed. Overall, the available data on maneb are inconsistent. Some findings suggest a synergist effect with paraquat. However, there is little compelling evidence that maneb alone causes selective dopaminergic neurotoxicity. Regarding other dithiocarbamates, some in vitro studies have demonstrated neurotoxicity. Zineb was shown to reduce viability of SH-SY5Y cells at concentrations above 100 µM (Jia and Misra 2007), and of ziram was toxic–SK-N-MC cells at concentrations above 1 µM (Wang et al. 2006).

The observations from experimental data of maneb are particularly interesting, because also the observations from case-control studies suggest a synergistic effect of paraquat and maneb (Fig. 2 h) (Costello et al. 2009).

### Pesticide screening

A recent study investigated the association between PD risk and pesticide-exposure in an approach that was referred to as pesticide-wide association study. They researchers subsequently screened 53 pesticides for toxicity in induced pluripotent stem cells (iPSC)-derived dopaminergic neurons using concentrations of 30 µM. Ten pesticides toxic effect, indicated by a reduction of TH-positive cells These findings were confirmed in dose-response experiment, where all ten pesticides exhibited toxicity at 6 µM, with some showing toxic effects at even lower concentrations. Among them, there were the organophosphate naled, the dinitroaniline trifluralin, the and the organochlorides endosulfan and dicofol (Paul et al. 2023).

### Regulatory status of pesticides associated with PD

In recent years, many pesticides associated with PD have been removed from the market in the United States and the European Union. The following paragraphs outline the regulatory status of selected pesticides linked–PD.

The Stockholm Convention, an international treaty established in 2021 in Stockholm, Sweden, aims to protect human health and the environment. Under this treaty, the production and use of numerous hazardous pesticides are prohibited among the member countries (Secretariat of the Stockholm Convention 2025a).

### Herbicides

#### Paraquat

Paraquat was initially approved in the EU in 2003. However this approval was annulled in July 2007, leading–complete market withdrawal by July 2008 (PAN Europe 2025a, 2025b). In contrast, the United States Environmental Protection Agency (EPA) maintained paraquat’s classification as “restricted-use” pesticide in 2021 (U.S. Environmental Protection Agency (EPA) 2025c).

#### Triazines

Atrazine was widely used from the 1950s. It was banned in Germany in 1991 and withdrawn from the EU market in 2004 (EUR-Lex 2004). Nonetheless, atrazine remains permitted in the US (U.S. Environmental Protection Agency (EPA) 2025a).

#### Glyphosate

Glyphosate is currently authorized in many regions globally, including the EU, where its approval extends until 2033 (European Commission 2023b) and the US (U.S. Environmental Protection Agency (EPA) 2025d).

### Insecticides

#### Rotenone

Rotenone was withdrawn from the German market in 1987 and banned in the EU in 2008 (German Federal Institute for RIsk Assessment 2012). However, it remains in use in some regions, including Norway and the U.S. (U.S. Environmental Protection Agency (EPA) 2021).

#### Pyrethroids

The approval of permethrin was withdrawn in the EU in 2000 (EUR-Lex 2000). Nevertheless, it is still permitted in countries such as the US, Canada, and Australia. Other pyrethroids remain authorized in the EU. For instance, deltamethrin was approved in 2003 and remains authorized approved under strict application conditions (European Commission 2024). Cypermethrin was approved in 2006 in the EU, with its current authorization valid until January 2029 (Euopean Commission 2021).

#### Organochlorines

Dieldrin was used extensively from the 1950 s to the 1970 s (American Chemical Association 2016). It was banned in Germany in 1972 (Umweltbundesamt 2022) and in the European Community in 1979 (EUR-Lex 1979). The US banned dieldrin in 1987 (Agency for Toxic Substances and Disease Registry 2011). Dieldrin, along with other organochlorides (e.g. heptachlor, lindane, methoxychlor) is listed for elimination under the Stockholm Convention (Secretariat of the Stockholm Convention 2025b).

#### Organophoshates

The EU approval of chlorpyrifos expired in January 2021 (EUR-Lex 2020). In the US, all tolerances for chlorpyrifos were revoked in 2021, but this decision was later overturned in court. In December 2024, the EPA again recommended revoking all tolerances, with a final decision still pending (U.S. Environmental Protection Agency (EPA) 2025b). In May 2025, chlorpyrifos, was added to the Stockholm convention’s elimination list (Secretariat of the Stockholm Convention 2025b). The EU authorization of dichlorvos was withdrawn in 2007 (European Commission 2007), though registration of dichlorvos-containing products remains possible in the U.S. (U.S. Environmental Protection Agency (EPA) 2016). Monocroptophos has been banned in the U.S since 1988(Export-Import Bank of the United States 2025) and was never officially approved in the EU.

### Fungicides

#### Dithiocarbamates

Maneb was previously authorized in the EU, but has been effectively banned since its approval expired in 2018 (European Commission 2019). Macozeb, another dithiocarbamates was permitted until its authorization was not renewed December 2020 (European Commission 2020). The approval for metiram expired in January 2024 (European Commission 2023a).

#### Benzimidazoles

Benomyl was permitted in Europe from 1970. However, all authoritations for benomyl-containing pesticides expired in 2003 (Euopean Commission 2002). In the U.S., benomyl was approved in 1969, all registrations were withdrawn by 2002 (US Government 2002).

## Discussion

PD is the second most common neurodegenerative disorder after Alzheimer’s disease (Balestrino and Schapira 2020). Epidemiological studies suggest elevated PD risk among individuals exposed to pesticides, and experimental data support this association by demonstrating that various pesticides exert dopaminergic toxicity in vitro and in vivo.

Notably, many of the implicated compounds, such as paraquat, triazines, rotenone, some pyrethroids, most organochlorides, organophosphates, dithiocarbamates, and benzimidazoles, are no longer approved for use in many regions, including the EU and the United States. However, individuals with past exposures remain at risk, as the long latency of PD means disease may develop decades after exposure.

In recognition of occupational risk, France classified PD as an occupational disease in specific high-risk populations over a decade ago (Elbaz and Moisan 2016). In Germany, the Federal Ministry of Labour and Social Affairs recommended recognition of “Parkinson’s syndrome caused by pesticides” as an occupational disease in March 2024, followed years of evaluation (Federal Ministry of Labour and Social Affairs 2024). A recognition allows affected individuals–receive support and compensation through the German Statutory Accidence Insurance (DGUV), even before formal inclusion of PD in the occupational diseases ordinance (Sozialversicherung für Landwirtschaft, Forsten und Gartenbau 2024).

Despite this regulatory process, establishing a causal link between pesticide exposure and PD remains challenging (German Federal Institute for RIsk Assessment 2023). Major obstacles include difficulties in accurately quantifying past pesticide exposure. Most epidemiological studies rely on self-reports via questionnaires (Galanaud et al. 2005; Elbaz et al. 2009; van der Mark et al. 2014; Furlong et al. 2015; Moisan et al. 2015; Hancock et al. 2008; Seidler et al. 1996). Recall accuracy deteriorates over time and often overestimates exposure by 1.2 to 1.4-fold (Fuhrimann et al. 2024). Users were frequently exposed–multiple compounds simultaneously (van der Mark et al. 2014), complicating identification of specific causal agents. Classification by functional class (i.e. herbicides, insecticides, or fungicides) is also problematic due–overlapping uses (van der Mark et al. 2014). Furthermore, many pesticides persist in the environment, accumulating in soil, plants, and animals (Rasool et al. 2022).

While some studies reported associations between pesticide use and PD risk, robust exposure-response data for specific compounds remain scarce. Even the largest case-control studies enrolled only a few hundred cases, limiting statistical power. Many implicated pesticides have been banned years ago, requiring reliance on retrospective exposure histories. Studies indicate that recall of pesticide exposure declines over time (Mueller et al. 2022), making it inherently difficult–determine whether pesticide exposure contributed–disease development, particularly when PD is diagnosed in later life. In addition–recall bias and the respective nature of the available data, valid biomarkers are lacking and confounding factors further complicate interpretation. Consequently, most evidence concerns “pesticides” in general. Due–lack of memory a distinction of pesticides is extremely challenging in retrospective analyses. Thus, the data individual functional groups are weak with heterogenous results, with data on individual compounds being even weaker. Despite the huge number of compounds used as pesticides, only paraquat (12), maneb (6), permethrin (6), benomyl (4), and diazinon (4), have been examined in more than four case-control. Yet none of these are still approve in the EU or U.S.

Data on acute pesticide poisoning in humans are sparse, mostly limited–case-reports. Only one study investigated the long-term effect of acute pesticide poisoning and found a modest increase in PD incidence (Chuang et al. 2017).

Experimental models, while informative, have their own limitations. Although certain pesticides exert dopaminergic toxicity in vitro and in vivo, acute exposure paradigms with high concentrations may not accurately reflect the chronic, low-level exposure patters typical in occupational settings. For instance, paraquat has been shown to elicit some effects only at concentrations around 10 mM (Richardson et al. 2005), far exceeding real-world exposure levels. Furthermore, cultured neurons lack complex physiological networks, and results are dependent on culture conditions and the presence or absence of glial cells (Falkenburger and Schulz 2006), thereby not taking metabolism of pesticide in whole organisms into account. Notably, for some pesticides, experimental data suggest toxicity to dopaminergic cells (e.g. atrazine), while epidemiological data could not convincingly demonstrate an association with PD risk, whereas for other pesticides, experimental data did not convincingly show dopaminergic toxicity (e.g. chlorpyrifos), while epidemiological studies revealed significant effects. This highlights the challenge to compare experimental data acquired in acute toxicity models with real-world exposure of human beings.

The present work has limitations itself. Despite attempts at comprehensive coverage, not all literature could be assessed. Overlapping case-control groups for many publications may be a possible source for a publication bias, although no systematic downgrading was applied. Retrospective study designs inherently limit evidence quality. Selection bias may have occurred, as only studies directly addressing PD and pesticides were included. Another approach would have been to assess all studies addressing the effects of pesticides on humans and evaluate, if these revealed an overrepresentation of PD as consequence of pesticide exposure. Although cohort or registry studies were reviewed, the focus was on cases-control studies, judged to provide the strongest evidence.

Age remains the strongest risk factor for PD, affecting over 1% of the population aged over 60 (Reeve et al. 2014), with incidence (Wattenbach et al. 2024) and prevalence (Pringsheim et al. 2014) rising with age. In the oldest age group, PD prevalence reaches approximately 4% (Tysnes and Storstein 2017). This demographic reality complicates efforts to distinguish environmental form age-related effects. Furthermore, some epidemiological suggest age-dependent pesticide effects with some reporting associations only in men over 65 (Elbaz et al. 2009) and others exclusively in individuals younger than 60 (Costello et al. 2009). This potential interaction between age and susceptibility to pesticides complicates efforts–disentangle the individual effects of age and pesticide exposure. In addition, it is well established that personal protective equipment (PPE) plays a crucial role in reducing pesticide exposure (Nguyen and Tsai 2024; DellaValle et al. 2012), and that the use of PPE has become more common in recent decades after regulatory changes in the 1970 s compared to the 1950 s and 1960 s (Bohme 2015).

Genetic susceptibility further complicates interpretation. Although the interaction between genetic variants and pesticide susceptibility is not yet fully understood, some studies suggest an increased risk of PD among individuals with specific genetic variants (Ritz et al. 2016), meaning individuals may have a higher PD risk despite a lower pesticide exposure burden than others.

Nevertheless, more thoroughly documentation of pesticide use would increase the data basis for future analyses and enable a better assessment of the risk of pesticides in the etiology of PD in the future. Changes of regulations and banning of many compounds used as pesticides, was an important step ro reduce the risk of environmental exposure–many pesticides. On the other hand, new compounds were permitted in the past years (e.g. cypermethrin in 2006 in the EU). A regulatory requirement for investing new compounds in PD models in the process of approval would be a possible measure for prevention in the future. Acute poisoning could be avoided by usage of appropriate protective gear. Therefore, pesticide applicators should be required to use this. However, chronic ingestion of pesticides by contaminated food is far more difficult to avoid.

In summary, while epidemiological and experimental evidence suggests a link between pesticide exposure and PD, numerous methodological limitations prevent definitive conclusions. The recent recognition of pesticide-induced Parkinsonism as an occupational disease in Germany and over a decade earlier in France reflects the growing body of evidence supporting this association. However, further research is needed, incorporating precise exposure assessment methods, long-term prospective studies, and experimental models that better reflect real-world human exposure. Future studies should also include biomarkers of exposure, genetic susceptibility factors, and mechanistic endpoints–provide a more comprehensive understanding of the role of pesticides in the pathogenesis of PD.

## Supplementary Information

Below is the link to the electronic supplementary material.

Supplementary Material 1.
